# An All-In-One Transcriptome-Based Assay to Identify Therapy-Guiding Genomic Aberrations in Nonsmall Cell Lung Cancer Patients

**DOI:** 10.3390/cancers12102843

**Published:** 2020-10-01

**Authors:** Jiacong Wei, Anna A. Rybczynska, Pei Meng, Martijn Terpstra, Ali Saber, Jantine Sietzema, Wim Timens, Ed Schuuring, T. Jeroen N. Hiltermann, Harry J. M. Groen, Anthonie J. van der Wekken, Anke van den Berg, Klaas Kok

**Affiliations:** 1 Department of Genetics, University Medical Centre Groningen, University of Groningen, 9700RB Groningen, The Netherlands; weijiacong@126.com (J.W.); a.a.rybczynska@rug.nl (A.A.R.); m.m.terpstra@umcg.nl (M.T.); 2 Department of Pathology, National Cancer Center/National Clinical Research Center for Cancer/Cancer Hospital, Chinese Academy of Medical Sciences and Peking Union Medical College, Beijing 100029, China; 3 Department of Pathology and Medical Biology, University Medical Centre Groningen, University of Groningen, 9700RB Groningen, The Netherlands; p.meng@umcg.nl (P.M.); a.saber@umcg.nl (A.S.); j.g.sietzema@umcg.nl (J.S.); w.timens@umcg.nl (W.T.); e.schuuring@umcg.nl (E.S.); a.van.den.berg01@umcg.nl (A.v.d.B.); 4 Department of Pathology, Collaborative and Creative Centre, Shantou University Medical College, Shantou 515063, Guangdong, China; 5 Department of Pulmonary Diseases, University Medical Centre Groningen, University of Groningen, 9700RB Groningen, The Netherlands; t.j.n.hiltermann@umcg.nl (T.J.N.H.); h.j.m.groen@umcg.nl (H.J.M.G.); a.j.van.der.wekken@umcg.nl (A.J.v.d.W.)

**Keywords:** RNA sequencing, non-small cell lung cancer, mutation, gene fusion, exon skipping

## Abstract

**Simple Summary:**

Treatment of patients diagnosed with advanced pulmonary adenocarcinoma depends on the presence of genomic aberrations that are targetable for a specific tyrosine kinase inhibitor. Subsequent treatment lines depend on presence of mutations that are associated with emerging resistance. These aberrations include a variety of gene activating mutations, including single nucleotide variants, small insertion-deletions, exon skipping events, and gene fusions. At this moment different assays are used to detect these aberrations in the clinic. In this paper we introduce a novel method that can detect these genomic alterations in a single, RNA-based, assay. The design of the all-in-one assay is flexible allowing addition of new targets in subsequent designs. We show that this all-in-one assay has a high accuracy even on formalin-fixed-paraffin-embedded tissue samples, making it readily applicable in a clinical diagnostic setting.

**Abstract:**

The number of genomic aberrations known to be relevant in making therapeutic decisions for non-small cell lung cancer patients has increased in the past decade. Multiple molecular tests are required to reliably establish the presence of these aberrations, which is challenging because available tissue specimens are generally small. To optimize diagnostic testing, we developed a transcriptome-based next-generation sequencing (NGS) assay based on single primed enrichment technology. We interrogated 11 cell lines, two patient-derived frozen biopsies, nine pleural effusion, and 29 formalin-fixed paraffin-embedded (FFPE) samples. All clinical samples were selected based on previously identified mutations at the DNA level in *EGFR, KRAS, ALK, PIK3CA, BRAF, AKT1, MET, NRAS,* or *ROS1* at the DNA level, or fusion genes at the chromosome level, or by aberrant protein expression of *ALK*, *ROS1*, *RET,* and *NTRK1*. A successful analysis is dependent on the number of unique reads and the RNA quality, as indicated by the DV200 value. In 27 out of 51 samples with >50 K unique reads and a DV200 >30, all 19 single nucleotide variants (SNVs)/small insertions and deletions (INDELs), three *MET* exon 14 skipping events, and 13 fusion gene transcripts were detected at the RNA level, giving a test accuracy of 100%. In summary, this lung-cancer-specific all-in-one transcriptome-based assay for the simultaneous detection of mutations and fusion genes is highly sensitive.

## 1. Introduction

Lung cancer accounts for approximately 27% of all cancer-related deaths worldwide [1]. The discovery of targetable driver genes in a subset of non-small cell lung cancer (NSCLC) patients has shaped personalized targeted therapies and prolonged patient survival [2,3,4,5]. Therapeutically relevant aberrations include, amongst others, activating mutations in *Epidermal Growth Factor Receptor* (*EGFR)*, *Kirsten Rat Sarcoma Oncogene* (*KRAS)*, *B-Raf Proto-Oncogene* (*BRAF),* and *MET Proto-Oncogene* (*MET)*, and fusion genes leading to activation of *Anaplastic Lymphoma Receptor Tyrosine Kinase (ALK)*, *ROS Proto-Oncogene 1* (*ROS1)*, *rearranged during transfection Proto-Oncogene* (*RET),* and *Neurotrophic Receptor Tyrosine Kinase 1 (NTRK1)*. Different diagnostic tests are required to reliably identify these aberrations in clinical settings, and the most commonly used techniques to detect these aberrations are targeted DNA sequencing for single nucleotide variants (SNVs) and small insertions and deletions (INDELs), fluorescence in situ hybridization (FISH) for chromosomal breaks, and immunohistochemistry (IHC) for aberrant expression of *ALK* and *ROS1* [6,7,8,9,10].

A recurrent problem, especially for advanced-stage NSCLC patients, is the small biopsy size in combination with the frequently low tumor content. This hampers comprehensive molecular testing using a combination of different tests to reliably screen for the presence of all types of clinically relevant genomic aberrations. To overcome this limitation, a comprehensive test to detect all aberrations in a single assay is needed. In several studies, a combination of DNA- and RNA-based next-generation sequencing (NGS) tests was applied, limiting the screening to two parallel tests [11,12,13,14,15]. In a study using a targeted RNA-based NGS test on frozen cytological samples from lung cancer and thyroid cancer, both fusion genes and mutations were simultaneously identified [16]. However, the currently available all-in-one approaches that cover all the different types of aberrations are not yet commonly applied in routine clinical settings.

In this study, we design a lung-cancer-specific targeted all-in-one transcriptome-based assay based on single primed enrichment technology (SPET) to simultaneously identify mutations, gene fusions, and exon skipping events. The assay covers all the gene loci that are currently relevant for selecting optimal targeted therapy in advanced stage NSCLC patients and does not require prior knowledge about the fusion gene partners. We tested the effectiveness of our comprehensive assay in samples with known aberrations, either based on the literature (i.e., cell lines) or on our routine molecular diagnostic test results. We specifically aimed to test its feasibility on formalin-fixed paraffin-embedded (FFPE) tissue samples.

## 2. Results

### 2.1. Sequencing Results

**Figure 1:**
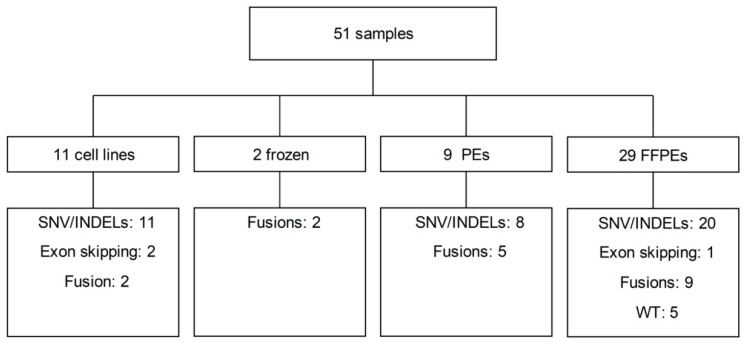
Schematic representation of the 51 samples included in our all-in-one transcriptome assay and the 60 known mutations. Shown are the number of samples for each source of tumor material. Lower boxes indicate the expected number of single nucleotide variants (SNVs)/insertions or deletions (INDELs); *MET* exon skipping mutations and fusion genes are indicated. WT: wild-type, samples without known mutations; PE, pleural effusions; FFPE, formalin-fixed paraffin-embedded tissue.

In total, we analyzed 51 samples derived from 37 patients and 11 cell lines that carried, in total, 60 known genomic alterations (Figure 1). All available RNA samples (*n* = 51) were subjected to our assay, irrespective of RNA quality and quantity, to gain insight into the overall performance of our assay. DV200 values were available for 42 samples. The median number of total reads obtained was 2.3 M (range: 1.6 to 5.1 M), and the median number of unique reads was 156 K (range: 1.2 to 1.3 M). For an overview of all quality-control data, see Table S1.

### 2.2. Detection of SNVs and INDELs

**Figure 2:**
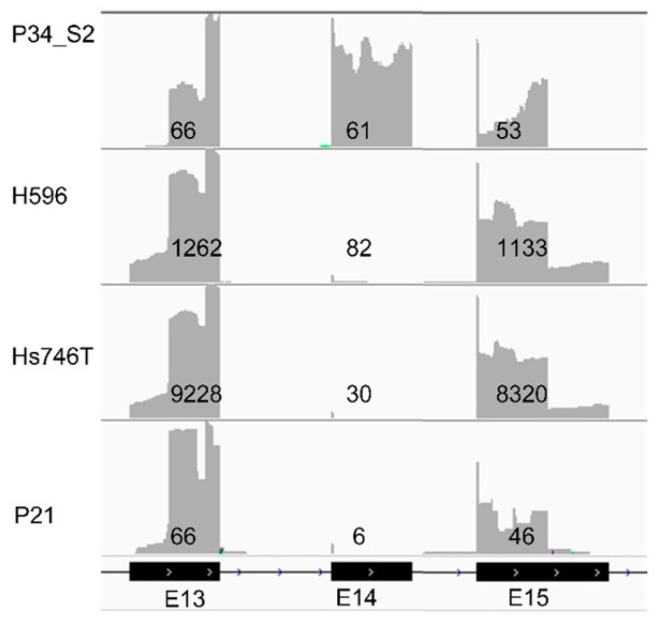
Integrated Genomics Viewer (IGV) screenshot of reads mapping to *MET* exons 13 to 15 for a randomly selected control sample (P34_S2) without *MET* exon 14 skipping, two cell lines H596 and Hs746, and one patient (P21) with known *MET* exon 14 skipping mutations. Numbers indicate the average coverage per exon.

Visual inspection of the aligned reads of our transcriptome assay in the Integrated Genomics Viewer (IGV 2.6.3, Cambridge, MA, USA) [17] revealed the presence of 34 of the 42 expected variants, with at least three mutant reads (Table 1). These included 25 SNVs, 6 INDELs, and the consequences of three *MET* exon 14 skipping mutations at the transcript level (Figure 2).

**Figure 3:**
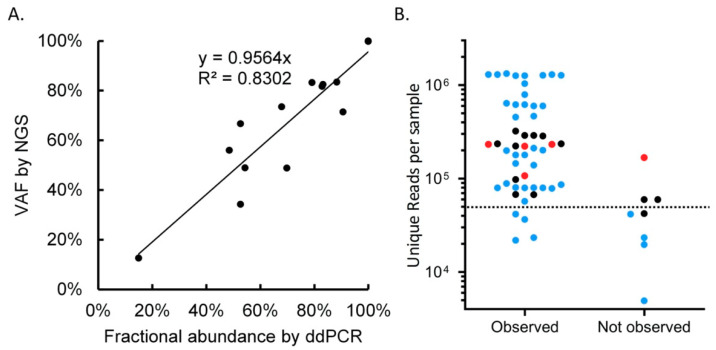
Validation by droplet digital (dd)PCR and threshold estimation for FFPE samples. (**A**) Comparison of the variant allele frequencies (VAFs) as detected by the all-in-one transcriptome-based assay and ddPCR. The Y-axis represents VAFs of the mutations, as assessed by our all-in-one next-generation sequencing (NGS) assay. The X-axis represents the fraction abundance calculated from ddPCR. (**B**) Overview of unique read counts (Y-axis) in samples for which we did and did not observe the genomic aberrations with our all-in-one transcriptome-based assay. •Blue dots indicate samples with DV200 above 30. •Red dots indicate samples with DV200 below 30. •Black dots indicate samples for which the DV200 value was not measured. Dashed line indicates the cut-off level of 50,000 unique reads.

Of the eight variants that we did not detect in IGV, we observed no mutant reads for seven cases and one mutant read for one case (Table 1). The total read depth was below 15 at the position of the expected mutation in all eight samples. To quantify the expression level of the wild-type and mutant alleles in the cases where we did not detect the mutation at the transcriptome level, we set up a highly sensitive RNA-based ddPCR assay. We first tested the efficiency of the ddPCR assay in 13 samples harboring 14 confirmed SNVs/INDELs using 1–8 ng of RNA input and detected all SNVs and INDELs. A good correlation was observed between the variant allele frequencies (VAFs) determined by our all-in-one assay and by the ddPCR assay using the same batch of RNA (R-squared 0.83; Figure 3A and Table S2). For six of the eight samples in which we did not observe the expected variant in our assay, RNA was available for ddPCR. Despite using a high RNA input (497 ng), only eight wild-type and no mutant *KRAS* p.G12A droplets were detected for patient P32, indicating a very low *KRAS* expression level. For the other five samples, we did observe mutant droplets with a frequency ranging from 10% to 71% using an RNA input ranging from 25 to 370 ng. The number of mutant droplets was still much lower than the numbers observed in the positive control samples, for which we used a much lower RNA input. This implies that the abundances of both the wild-type and mutant transcripts were below the detection limit of our assay, due to either poor RNA quality, low expression of the gene, or insufficient unique reads in all six samples.

Next, we tested the performance of the inhouse pipeline in calling the variants observed by IGV 2.6.3. The three *MET* exon 14 skipping events were excluded as our transcriptome-based assay does not allow the detection of intronic variants. We analyzed all 51 samples with our pipeline and were able to call 28 of the 31 SNVs and INDELs observed by IGV. The three variants that were observed in IGV but not called by the pipeline were (1) *AKT Serine/Threonine Kinase 1 (AKT1)* p.E17K in P35, with 81 mutant reads out of 292 total reads, (2) *ALK* p.G1269A in P13, with 44 mutant reads out of 240 total reads, and (3) *EGFR* E19 DEL in P22, with 13 mutant reads out of 38 total reads. A second variant caller, Freebayes, did call the *AKT1* p.E17K mutation but not the other two. The most likely reason that our pipeline did not call the *EGFR* E19 DEL is the improper alignment of part of the mutant reads (Figure S1). In the samples with confirmed *EGFR* E19 DELs, the number of reads with the deletion ranged from 20 to 311 in IGV, whereas the reported read counts ranged from 8 to 128 according to the pipeline. Apparently, our pipeline missed a subset of the reads containing the *EGFR* E19 DEL due to improper alignment or short read length. It is unclear why our pipeline did not call the other two variants.

In addition to the expected mutations, we detected four novel mutations (Table 1). Three of them were frameshift mutations (*NRAS* p.G15fs, *BRAF* p.Y472fs, and *KRAS* p.L56fs) that are not included in the molecular diagnostic reports because they are, as yet, not relevant in making therapeutic decisions. The fourth mutation was an *EGFR* p.V834L mutation observed in an FFPE sample (P03), in which we also detected a *KRAS* p.G12A mutation. The *KRAS* mutation was reported in the molecular diagnostics, whereas the *EGFR* mutation was not observed. Reanalysis of the same RNA sample with our SPET assay did not reveal this uncommon *EGFR* variant, leading us to conclude that this was a false-positive observation.

### 2.3. Fusion Gene Detection

We identified fusion transcripts for 13 of the 18 fusions reported by diagnostic tests (clinical samples) or literature (cell lines) using two fusion detection pipelines. No additional chimeric transcripts were identified. During visual inspection of the aligned reads in IGV, we observed partly unaligned reads for three additional samples. BLAST of the unaligned sequences indicated the presence of fusion transcripts that were not called by the two pipelines. Thus, of the 18 expected fusion transcripts, we confirmed 16 with our all-in-one transcriptome assay (11 *ALK*, three *ROS1*, one *RET,* and one *NTRK1*; Table 2). In addition to the detection of the target fusion genes, our assay also pinpointed the intron in which the break occurred and the fusion partner in all cases. Ten of the 11 *ALK* fusion transcripts correspond to previously published fusion transcripts: seven *Echinoderm Microtubule Associated Protein Like 4* (EML4)_E6-ALK_E20, one *Dynactin Subunit 1* (DCTN1)_E26-ALK_E20, and one *Kinesin Family Member 5B* (KIF5B)_E24-ALK_E20. For P36, we observed an uncommon breakpoint region located in intron 18 of the *ALK* gene that resulted in an EML4_E6-ALK_E18 fusion transcript. In P42, a novel *ALK* fusion transcript, *Myosin Phosphatase Rho Interacting Protein* (MPRIP)_E21-ALK_E20, was identified. The five non-EML4_ALK fusions were defined as *Ezrin* (EZR)_E10-ROS1_E34 in two cases, CD74_E6-ROS1_E34 in one case, KIF5B_E15-RET_E12 in one case, and a *Tropomyosin 3* (TPM3)_E7-N*eurotrophic Receptor Tyrosine Kinase 1* (NTRK1)_E9 in one case.

For two FISH-break and/or IHC-positive cases, no fusion gene transcripts were identified by our assay. P07 was scored as positive based on both *ALK* IHC and FISH, but no fusion transcript was observed with our transcriptome assay. Of note, we also missed the SNV in *ALK* for this patient due to the low total read count. For P08, *RET* was scored positive based on a FISH break pattern in 27% of the cells (2% true split and 25% extra red signals), while an *ALK* break was seen in 63% of the cells. With our assay, we only observed *ALK* fusion transcripts and no *RET* fusion transcripts.

As an independent validation, we applied NanoString to detect the fusion gene transcripts. We validated the assays on five confirmed cases and were able to identify the expected fusion transcripts by NanoString (Table S3). We next examined the two cases in which we did not find the expected fusion transcripts using our all-in-one assay. For P07, NanoString detected an EML4_E6-ALK_E20 fusion transcript. In P08, NanoString detected the *ALK*, but not the *RET* fusion transcript, consistent with our all-in-one assay. Thus, the negative result of our all-in-one assay was consistent with NanoString for one case.

As a previous study showed a potential association between survival on TKI treatment and the fusion gene partner [18], we also analyzed progression-free survival (PFS) in relation to the fusion partner of the nine *ALK*-positive patients (Table S4). Five patients with the canonical EML4_E6-ALK_E20 fusion transcripts were treated with crizotinib and had PFS of 6, 8, 9, 14, and 15 months. Patient P36, with an EML4_E6-ALK_E18 fusion gene, had PFS of 24 months. Patient P42, with MPRIP_E21-ALK_E20, had PFS of 8 months. Patient P08, with a KIF5B_E24-ALK_E20 fusion gene, had PFS of 19 months. Patient P14, with DCTN1_E26-ALK_E20 fusion transcript, did not respond to crizotinib, and treatment was changed to alectinib, again with no tumor response.

### 2.4. RNA Input Limit

It is challenging to set a clear RNA input limit for transcriptome-based assays. The tumor content of the sample and the expression level of the genes in nontumor cells are just two of the variables that play a role in setting the minimum amount of RNA needed for the assay. Despite these issues, we tried to get some insight into the detection limit of our assay. Eight samples were resequenced with an 8-fold lower library input. As a result, the number of unique reads for these samples decreased (Table S5). In all eight cases, the expected aberrations were again successfully called by the pipeline, with VAFs similar to those observed under standard conditions (Table S5). In addition, we repeated library preparation for three RNA samples using 4-fold and 20-fold lower RNA input. For two cases, this resulted in a 3- and 4-fold decrease of the number of unique reads with a 4-fold lower RNA input, and 5- and 12-fold decrease with 20-fold lower RNA input. For the third case, the pattern was less consistent for the 4-fold lower RNA input library (Table S6). Again, we were able to detect the expected variants for all samples irrespective of the RNA input. This indicates that a total RNA input of as low as 10 ng for library preparation is feasible for samples with sufficient RNA quality and high tumor cell content.

### 2.5. Quality Criteria for Successful Mutation Detection

We next established quality criteria for the successful detection of mutations. Without setting any quality criteria, the overall sensitivity of the assay was 87% (Table 3 and Table S7). The main factor for the successful identification of aberrations is the number of unique reads obtained in the NGS analysis (Table 3 and Figure 3B). To identify factors associated with the percentage of unique reads, we performed a univariable linear regression analysis. A significant correlation was observed with panel design version (1, 2, or 3), material type (FFPE, non-FFPE), RNA input, DV200, and the number of cycles used to amplify the library. In a multivariable analysis, material type and DV200 remained significant (Table S8). As FFPE is the standard material type in a routine diagnostic setting, we decided to reanalyze the sensitivity of our assay using a threshold of 50 K unique reads in combination with a DV200 threshold of 30 as quality criteria. In addition to the good quality samples, 13 FFPE samples fulfilled these quality criteria (Figure 3B and Table 3). When both criteria were applied, e.g., DV200 >30 and unique reads >50 K, all 35 expected variants were detected, leading to a sensitivity of 100% (Table 3). In the analyses of all the hotspots for which our series of samples were supposed to be negative (including the five nonmutated NSCLC cases), we had one false-positive observation. This also indicates that the specificity of the assay is close to 100%.

## 3. Discussion

The currently used tests to select therapy for advanced-stage NSCLC patients include sequencing-based methods to detect mutations in hotspot regions, FISH techniques to detect chromosomal breaks, and IHC to detect aberrant protein expression. A major advantage of the targeted transcriptome-based sequencing assay we report here is that it can efficiently pinpoint all types of somatic mutations that result in an aberrant transcript in a single test. As we interrogate the transcriptome, our assay also provides information on the expression of mutant alleles. In addition, our assay provides information on fusion partners and shows the consequence of *MET* exon skipping mutations at the transcript level. By setting quality criteria for both RNA (DV200 >30) and the total number of unique reads (>50 K reads), our assay identified all the expected mutations at the transcriptome level, with almost no false-positives, and thus reached an accuracy of close to 100%. The application of these two criteria to our FFPE samples would have resulted in the exclusion of 8 out of 21 FFPE samples for which DV200 data were available. For four of these cases, the DV200 value was too low, whereas for the other four samples, the unique read counts were too low. In a diagnostic setting, a new tissue sample would have been requested for all eight cases. The main reason for potential dropout in a routine setting will be related to the availability of sufficient tissue in combination with tumor cell content and RNA quality. The use of freshly prepared FFPE blocks in combination with the macrodissection of tumor-cell-rich regions, which is standard procedure in diagnostic settings, will most likely increase the number of successfully analyzed samples. We expect that the dropout frequency for fresh FFPE blocks will be similar to the current dropout using DNA-based NGS assays, which are also dependent on the availability of sufficient tissue in combination with tumor content and quality of the isolated DNA. Thus, our all-in-one transcriptome-based assay is expected to have an overall good performance on clinical FFPE samples using predefined quality criteria. In a related study, we successfully used input RNA amounts of as low as 150 ng, isolated from fresh FFPE material. A limited number of experiments performed in our current study indicated that with fresh frozen material, even 10-ng quantities will suffice (Table S6).

For fusion genes, both the juxtaposed exons of the target gene as well as the specific fusion partners were identified. In addition to the common fusion products, we found several uncommon fusion partners. The MPRIP_E21-ALK_E20 fusion product has only been reported once in a conference abstract [19]. A second uncommon fusion transcript involving *ALK* had a break in intron 18 of the *ALK* gene, and the resulting EML4_E6-ALK_E18 fusion transcript has only been reported once [20]. Detailed knowledge of the fusion partner and/or breakpoints could have clinical implications. In a recent study on *ALK* FISH-positive lung cancer, patients with canonical fusion partners involving *EML4* were found to have better overall survival (20.6 months vs. 5.4 months, *p* < 0.01) than those with noncanonical *ALK* fusions [18]. In our study of a limited number of patients, the PFS of patients with canonical and noncanonical breaks were similar. Still, the implementation of techniques to identify the fusion gene partner may become important in routine diagnostics in situations where knowledge about the fusion gene does predict drug response. These studies further underscore the importance of implementing transcriptome-based tests in the diagnostic setting.

At the DNA level, several specific mutations in introns 13 and 14 of *MET* have been linked to exon 14 skipping at the transcript level, but for other novel mutations, it remains unclear whether this indeed leads to *MET* exon 14 skipping [21]. Detection of *MET* exon skipping using a transcriptome-based NGS method, as described in this study, directly measures the consequence of the mutation even though the actual mutation causing the exon skipping event will not be identified.

Another potential application of our assay might be the assessment of overexpression of specific genes and its use as an indirect method to identify gene amplifications. This is relevant for *MET* and *ERBB2*, as amplification of their gene loci has been reported as a resistance mechanism for targeted TKI treatment. Implementation of this application will require additional validation experiments that are beyond the scope of the current study. Application of an all-in-one transcriptome-based assay maximizes the success rate of detecting aberrations, especially for lung cancer biopsies with generally limited tissue volume. Previously, a SPET-based method has been shown to successfully identify fusion transcripts [14]. In current molecular diagnostic settings, a few methods are available to simultaneously identify SNVs, INDELs, exon skipping, and gene fusions. A bait-based library enrichment method used to detect SNVs, INDELs, translocations, inversions, and copy number variations (CNVs) in FFPE-derived DNA confirmed the presence of all 34 known aberrations [22]. In addition, they identified ALK fusions, including the fusion partner in six out of seven ALK IHC-positive cases. In another study, 100% concordance was found between 10 paired FFPE and frozen biopsy cases [16]. Nowadays, a few commercial platforms use RNA to detect gene fusions. A comparison between their performances has very recently been published [23]. Several platforms combine the RNA-based part with a DNA-based procedure to enable the detection of SNVs and small indels. Our assay competes with currently available commercial panels, which have a much larger number of target genes. The large commercial panels require higher total numbers of reads per sample, which effect the number of samples that can be pooled in one sequence run, and thus increase the per-sample costs. Moreover, our assay can easily be adapted when the number of therapy-guiding mutations increases. An additional advantage of our assay is that it not only efficiently identifies various types of driver/actionable mutations but, at the same time, verifies the expression of the mutant alleles in the tumor cells. The SPET strategy, allowing target enrichment using a single gene-specific landing-probe designed close to the mutational hotspot, can give clinically relevant results even for samples with highly fragmented RNA. The use of UMIs allows reliable filtering for unique reads and, thereby, allows reliable identification of all variants, including unknown fusion partners. Although the use of RNA instead of DNA may be regarded as challenging, RNA-based assays are being used more regularly in diagnostic settings in academic hospitals. As several steps of the procedure can be automated, the amount of hands-on time will be similar to currently used DNA-based tests. Overall, we anticipate that our test will be competitive with current techniques for both hands-on time and costs.

To further assess the applicability of our all-in-one assay in a clinical diagnostic setting, a prospective study on routinely requested lung tumor samples should be carried out.

## 4. Materials and Methods

### 4.1. Sample Information

We included seven lung-cancer-derived cell lines (A549, H1299, H1650, H1975, H2228, H596, H820), four cell lines derived from other cancer types (KM12, HCT116, Hs746T, KOPN-8) with specific genomic aberrations relevant for lung cancer [24,25,26,27,28,29,30,31,32,33,34,35,36], and 40 tissue samples of patients with known genomic aberrations identified between 2011 and 2017 (Figure 1, Table S1) and five FFPE tissue samples without known mutations. Cell line KOPN-8 was obtained from the German Collection of Microorganisms and Cell Lines (DSMZ). KM12 was obtained from the National Cancer Institute (Boston, MA, USA). All additional cell lines were obtained from the American Type Culture Collection (ATCC). Cell lines were cultured in RPMI-1640 supplemented with 10% fetal bovine serum (FBS) and 5% penicillin/streptomycin, following standard culturing protocols. The origin of the patient samples was pleural effusion (PE; nine samples from nine patients), frozen tissue (two samples from two patients), and FFPE tissue (29 samples from 28 patients).

In total, these 51 samples covered 60 known variants, i.e., 39 SNVs/INDELs, three *MET* exon skipping, and 18 fusion genes (Figure 1). Five lung tumor tissue samples without molecular aberrations, according to the routine molecular tests, were included as nonmutated NSCLC cases. All patient samples were obtained from the UMCG pathology biobank and were anonymized for the investigators. The study protocol is consistent with the Research Code of the University Medical Centre Groningen (https://www.umcg.nl/SiteCollectionDocuments/English/Researchcode/umcg-research-code-2018-en.pdf, accessed on 22 January 2020) and national ethical and professional guidelines (“Code of conduct; Dutch federation of biomedical scientific societies”, htttp://www.federa.org/codes-conduct, accessed on 1 June 2019).

### 4.2. RNA Isolation

RNA from cell lines and PE samples was isolated using the GeneJET RNA Purification Kit (Thermo Fisher Scientific, Waltham, MA, USA), and RNA from frozen tissue samples was isolated using a TRIzol (Invitrogen, Waltham, MA, USA)-based standard laboratory protocol, including a phase separation step with chloroform and subsequent RNA precipitation with isopropanol. The RNeasy FFPE Kit (QIAGEN GmbH, Hilden, Germany) was used for RNA isolation from total tissue sections of FFPE tissue samples without enrichment of tumor-cell-rich areas. For all kits, isolation procedures were done according to the manufacturer’s protocol. The quantity and quality of the RNA samples were analyzed using nanodrop (Thermo Fisher Scientific, Waltham, MA, USA) and Fragment Analyzer (Advanced Analytical, Armes, IA, USA), respectively. The obtained DV200 value obtained indicates the percentage of RNA fragments that are longer than 200 nucleotides (Table S1).

### 4.3. Design of All-In-One Lung Cancer Assay

Our assay is based on the single primer enrichment technology developed by NuGEN^©^ (SPET, United States Patent 9,650,628; San Carlos, CA, USA). The target region for the assay was designed to cover all clinically relevant genomic aberrations (Table S9). For mutation hotspots, landing probes were designed within 50 nucleotides upstream and downstream of each target region. Target regions included in the assay were based on the routinely used custom-designed diagnostic amplicon-based panel: *BRAF* (codons 466, 499, and 600), *EGFR* (codons 790, 858, exon 19 deletion (E19 DEL) regions, and all mutated codons in exons 18–21), *Phosphatidylinositol-4,5-Bisphosphate 3-Kinase Catalytic Subunit Alpha* (*PIK3CA;* codons 442, 545, and 1047), *KRAS* (codons 12, 13, and 61), *NRAS* (codons 12, 13, and 61), *Discoidin Domain Receptor Tyrosine Kinase 2* (*DDR2*; codon 768), *AKT1* (codon 17), *Erb-B2 Receptor Tyrosine Kinase 2* (*ERBB2*; exon 20), *Mitogen-Activated Protein Kinase 1* (*MAP2K1*; codons 56, 57, and 67), and the tyrosine kinase domains of *ALK* and *ROS1*. For fusion genes routinely tested by IHC or FISH in the molecular diagnostics, i.e., *ALK*, *ROS1*, *RET,* and *NTRK1,* and the most frequently observed fusion partner genes, we included the relevant landing probes from the Ovation Fusion Panel Target Enrichment System kit (NuGEN Technologies, San Carlos, CA, USA) [14]. These landing probes were close to the boundaries of the exons facing towards the flanking up- and downstream exons. In addition, landing probes were designed at the boundary of exon 13, facing exon 14, and at the boundary of exon 15, facing exon 14 of the *MET* gene, to detect exon 14 skipping events. Finally, we added landing probes for a selection of housekeeping genes to serve as internal quality controls. Due to the small target region of our assay, it does not allow the detection of the tumor mutational burden (TMB).

Over the course of this study, we developed three versions of our design. Minor changes in landing probe regions were subsequently made to optimize coverage of the hotspot regions, whereas the target regions remained the same. Furthermore, the highly expressed housekeeping genes added in Design 1 were replaced by less abundantly expressed housekeeping genes in later designs. Landing probes in genes relevant for immunotherapy were added in the third design, but these were not analyzed in detail in this study. The number of landing probes for each design is indicated in Table S9. The genomic locations of the landing probes are indicated in the File S1.

### 4.4. Library Preparation

We aimed for an RNA input of 200 ng for non-FFPE and 500 ng for FFPE samples for library preparation, as measured by nanodrop, and cDNA synthesis was done using the cDNA Module for Target enrichment (NuGEN), following the recommendations of the manufacturer. Library preparation for the SPET procedure was done according to the protocol provided by the manufacturer NuGEN^©^ (for a graphical outline, see https://ww.nugen.com/products/technology) and described in detail elsewhere [14,37]. Briefly, after ds-cDNA synthesis, adaptors containing an 8-nt sample-specific barcode, a 6-nt unique molecular identifier, and a universal forward primer were ligated to the fragments. The resulting fragments were denatured, and landing probes containing the universal reverse primer were hybridized overnight, followed by an extension step. Subsequently, a test qPCR was done to determine the optimal number of cycles for library amplification. The number of cycles used for the library amplification was 0 to 4 above the cycle threshold determined by the test qPCR, as recommended by the manufacturer. After amplification, TapeStation measurement and/or Kapa qPCR were done to determine the molarity of the library. Eight or sixteen libraries were mixed in equimolar amounts and subjected to NGS on a MiSeq platform (Illumina, San Diego, CA, USA), with a 150 bp paired-end sequencing protocol provided by the manufacturer. Adaptor and primer sequences are shown in Figure S2.

For eight samples, the library was sequenced a second time with 1/8 of the standard input. For three high-quality RNA samples, libraries were prepared using three different amounts of RNA input: 200, 50, and 10 ng (Table S7).

### 4.5. NGS Data Analysis

The FASTQ files were processed with an inhouse pipeline. Alignment of reads was done using Hisat2, and Genome Analysis Toolkit (GATK 3.8.0, Cambridge, MA, USA) human genome reference build GRCh37, with decoys from the GATK bundle [38,39]. Picard Tools was used for format conversion and marking duplicates, including the unique molecular identifier information of the reads. We carried out a manual check using the IGV browser V2.6.3 for all known SNVs and INDELs, starting from the aligned reads. In addition, we designed a pipeline for variant detection. Haplotype Caller was used for the integrated calling of the variants for all samples. Variants were annotated using SnpEff/SnpSift with the Ensembl release 75 gene annotations and the dbNSFP2.7, dbsnp 138, Cosmic v72, 1000 genomes phase 3, and the ExAC 0.3 databases [40,41,42]. Our variant-calling pipelines for SNVs and INDELs are an adaptation of the GATK workflow and use Molgenis Compute [43] as the workflow management software. The data were filtered for quality metrics similar to GATK recommendations, using custom filters for population frequency and variant effect. Synonymous mutations, variants present in the 1000 human genome project at a frequency of more than 2%, variants with less than three altered read counts or a variant allele frequency (VAF) less than 5%, and variants with CADD scores less than 20 were filtered out [44]. Fusion gene detection was done with Fusion Catcher and Strand NGS software (Strand Genomics, San Francisco, USA) [45]. We focused on fusion gene analyses on *ALK*, *ROS1*, *RET,* and *NTRK1*. We only report inframe fusion transcripts with the tyrosine kinase domain of the indicated genes as well as those in which the sum of spanning and splitting read counts was at least five. Recurrent mutations that exclusively occur at the end of the reads were excluded because these most likely represent technical artefacts. Reads that could only be aligned to part of the fusion gene region, i.e., indicative of a fusion gene breakpoint split read, were subjected to BLAT analysis to identify the fusion gene partner [46].

### 4.6. Detection of Fusion Gene Transcripts by NanoString

Using the Lung Gene Fusion Panel (NanoString Technologies, Seattle, WA, USA), we detected fusion transcripts in *ALK*, *RET,* and *ROS1*. A total amount of 100–200 ng RNA was hybridized overnight following the manufacturer’s protocol. The next day, samples were loaded on streptavidin-coated cartridges and analyzed on an nCounter^®^ SPRINT Profiler (NanoString technologies). The raw barcode counts were background-adjusted with a truncated Poisson correction using negative-control spikes and normalized relative to the positive-control spikes. Samples with good hybridization quality, as determined by good signals for housekeeping genes and counts below 30 for negative controls, were included for calling fusion transcripts. A *t*-test between the 3′ and 5′ probe counts was applied to identify imbalance probes. The presence of a fusion transcript is defined as positive based on the following criteria: the *p*-value is <0.01 for the 3′ and 5′ count difference, the 3′/5′ ratio and the 3′/negative control count ratio are both >1.5, the absolute counts of fusion-specific probes are >20 (*ALK* and *ROS1*) or >30 (*RET*), and counts of the fusion-specific probe are >2× SD of the mean probe count across the gene, except for the outlier counts, which are above the upper Tukey fence (Q3 + 1.5*IQR).

### 4.7. Variant Detection by Droplet Digital (dd) PCR

We applied ddPCR (Bio-Rad, Hercules, CA, USA) on cDNA to quantify expression of the mutant allele of the most commonly observed mutations, e.g., T790M, L858R, E19 DEL in *EGFR,* and G12A, G12D, G12F in *KRAS*. For RNA samples from cell lines, cDNA was synthesized with the RevertAid H Minus First Strand cDNA Synthesis Kit (Thermo Scientific, Madison, WI, USA). For RNA from clinical FFPE tissues and pleural effusions, cDNA was synthesized with the iScript cDNA Synthesis Kit (Bio-Rad). Negative and positive control samples were included for each variant in all experiments. For good quality samples, RNA input was 1–2 ng. For other samples, the input varied between 5 and 479 ng. Reaction mixes included 11 μL ddPCR Supermix for probes and 1 μL mutation assay in a final volume of 22 μL. Droplets were generated using the QX100 droplet generator after the addition of 70 μL droplet generation oil (Bio-Rad). PCR was performed on a T100 Thermal Cycler (Bio-Rad), using the following PCR conditions: 95 °C for 10 min, 39 cycles of 95 °C for 30 s, 59 °C for *KRAS* or 55 °C for *EGFR* and *ALK* for 60 s, 72 °C for 15 s, 98 °C for 10 min, followed by a cooling down to 4 °C. The temperature ramp change was 2 °C per second for all steps. Droplets were counted on a QX-100 droplet reader (Bio-Rad), and data were analyzed by Quantasoft software version 1.6.6 (Quantasoft, Prague, Czech Republic) for detection of FAM and HEX signals. For *EGFR* T790M, the forward and reverse primers were 3′-CAAGGAAATCCTCGATGAAGCC-5′ and 3′-GTCTTTGTGTTCCCGGACATAGT-5′ with a HEX-labelled wild-type probe 3′-ATGAGCTGCGTGATGAG-5′ and a FAM-labelled mutant probe 3-ATGAGCTGCATGATGAG-5′. For *EGFR* L858R, the forward and reverse primers were 3′-GCAGCATGTCAAGATCACAGATT-5′ and 3′-CATCCACTTGATAGGCACTTTGC-5′ with a HEX-labelled wild-type probe 3′-AGTTTGGCCAGCCCAA-5′ and a FAM-labelled mutant probe 3′-AGTTTGGCCCGCCCAA-5′. For *EGFR* exon 19 deletions, primers used were 3′-GTGAGAAAGTTAAAATTCCCGTC-5′ and 3′-TGGCCATCACGTAGGCTTC-5′ with a FAM-labelled probe covering the deletion part of exon 19 3′-AAGGAATTAAGAGAAGCAACATCTCC-5‘ and a wild-type HEX control probe upstream of the commonly deleted region 3′-ATCGAGGATTTCCTTGTTGGCT-5′. Primer and probe details of *KRAS* are according to the literature [47]. Data were analyzed using Bio-Rad QuantaSoft™ Analysis Pro (Bio-Rad). The threshold for the mutant droplet signal was set manually. The number of mutant and wild-type copies was estimated from the Poisson distribution, as indicated by the manufacturer. Fractional abundance was calculated using mutant copies divided by the sum of mutant and wild-type copies.

### 4.8. Statistics

To estimate which sample and test conditions were important for an optimal result of the all-in-one transcriptome test, linear regression analysis was performed using SPSS (version 23.0, IBM, Armonk, NY, USA). The uniquely aligned sequencing reads were the dependent variable. The independent variables were design version, tissue origin (FFPE or non-FFPE), RNA input, DV200, and the number of PCR cycles used for library preparation. Parameters with *p* < 0.1 in the univariable analyses were further included in the multivariable analysis. To study the relationship between the ALK fusion partner and survival, PFS was calculated from the date that treatment was started to the date of progressive disease on CT imaging, according to the Response Evaluation Criteria in Solid Tumors (RECIST v1.1) [48].

## 5. Conclusions

This study demonstrates the feasibility and technical validity of a targeted all-in-one transcriptome-based assay for the simultaneous detection of mutations and fusions in relatively small FFPE tissue biopsies. We expect that for routine diagnostic testing using 150 ng RNA isolated from recent FFPE tissue samples in combination with an enrichment step to increase tumor cell content, the success rate will be similar to currently used diagnostic tests, with an overall accuracy close to 100%. To get the highest performance, it is important to set minimum requirements for RNA quality and total unique reads, similar to the standard procedures for DNA-based NGS diagnostic tests.

## Figures and Tables

**Table 1:** Overview of SNV/INDEL samples analyzed by the all-in-one transcriptome-based assay and summary of the results.

Sample ID	Origin	DV200	Known Variants Detected at DNA Level			Results of All-In-One Transcriptome-Based Assay ^a^				
			Gene	Amino Acid Change	MD Test or Reference	Tool	Mutant Reads	Total Reads	VAF ^c^	Status
Variants Known at DNA Level										
P35	PE	81	*AKT1*	p.E17K	NGS	IGV	81	292	28%	confirmed
P13	PE	89	*ALK*	p.G1269A	NGS	IGV	44	240	18%	confirmed
P13	PE	89	*ALK*	p.I1171N	NGS	IGV and Pipeline	331	336	99%	confirmed
P07	FFPE	65	*ALK*	p.L1196M	NGS	IGV	1	3	33%	not confirmed
P35	PE	81	*BRAF*	p.V600E	NGS	IGV and Pipeline	31	60	52%	confirmed
P25	FFPE	71	*BRAF*	p.V600E	NGS	IGV and Pipeline	33	46	72%	confirmed
H1650	cell line	nd	*EGFR*	p.E746_A750del	NGS	IGV and Pipeline	46	76	61%	confirmed
H1975	cell line	nd	*EGFR*	p.T790M	NGS	IGV and Pipeline	347	425	82%	confirmed
H1975	cell line	nd	*EGFR*	p.L858R	NGS	IGV and Pipeline	564	684	82%	confirmed
H820	cell line	99	*EGFR*	p.E746_A750del	NGS	IGV and Pipeline	80	606	13%	confirmed
H820	cell line	99	*EGFR*	p.T790M	NGS	IGV and Pipeline	127	660	19%	confirmed
P04_S2	PE	88	*EGFR*	p.L858R	NGS	IGV and Pipeline	4661	4931	95%	confirmed
P05	PE	17	*EGFR*	p.E746_A750del	22	IGV	0	0		not confirmed
P04_S1	FFPE	26	*EGFR*	p.L858R	19,22	IGV and Pipeline	69	72	96%	confirmed
P06	FFPE	37	*EGFR*	p.L747_P753delinsS	22	IGV and Pipeline	8	17	47%	confirmed
P06	FFPE	37	*EGFR*	p.T790M	19,22	IGV	0	15	0%	not confirmed
P15	FFPE	40	*EGFR*	p.E746_A750del	NGS	IGV and Pipeline	51	76	67%	confirmed
P15	FFPE	40	*EGFR*	p.T790M	NGS	IGV and Pipeline	22	88	25%	confirmed
P17	FFPE	57	*EGFR*	p.E746_A750del	NGS	IGV and Pipeline	128	182	70%	confirmed
P17	FFPE	57	*EGFR*	p.T790M	NGS	IGV and Pipeline	62	127	49%	confirmed
P22	FFPE	69	*EGFR*	p.E746_A750del	19,22	IGV	13	38	34%	confirmed
P26	FFPE	nd	*EGFR*	p.L858R	NGS	IGV	0	6	0%	not confirmed
A549	cell line	nd	*KRAS*	p.G12S	18	IGV and Pipeline	512	513	100%	confirmed
HCT116	cell line	nd	*KRAS*	p.G13D	24	IGV and Pipeline	223	456	49%	confirmed
KOPN-8	cell line	99	*KRAS*	p.G12D	29	IGV and Pipeline	99	177	56%	confirmed
P01	PE	90	*KRAS*	p.G12D	NGS	IGV and Pipeline	14	111	13%	confirmed
P03	FFPE	nd	*KRAS*	p.G12A	NGS	IGV and Pipeline	8	8	100%	confirmed
P23	FFPE	44	*KRAS*	p.G12C	NGS	IGV	0	1		not confirmed
P28	FFPE	38	*KRAS*	p.G12A	NGS	IGV and Pipeline	8	22	36%	confirmed
P31	FFPE	66	*KRAS*	p.Q61H	NGS	IGV and Pipeline	60	123	49%	confirmed
P39	FFPE	65	*KRAS*	p.G12D	NGS	IGV and Pipeline	8	12	67%	confirmed
P40	FFPE	68	*KRAS*	p.G12F	NGS	IGV	0	1		not confirmed
P32	FFPE	32	*KRAS*	p.G12D	NGS	IGV	0	2		not confirmed
H596	cell line	nd	*MET*	Exon skipping mut.	27	IGV	1116	1196 ^b^	93%	confirmed
Hs746T	cell line	97	*MET*	Exon skipping mut.	30	IGV	8744	8774 ^b^	100%	confirmed
P21	FFPE	34	*MET*	Exon skipping mut.	NGS	IGV	50	56 ^b^	89%	confirmed
H1299	cell line	nd	*NRAS*	p.Q61K	20	IGV and Pipeline	1107	2549	43%	confirmed
H596	cell line	nd	*PIK3CA*	p.E545K	28	IGV and Pipeline	156	330	47%	confirmed
HCT116	cell line	nd	*PIK3CA*	p.H1047R	25	IGV and Pipeline	69	115	60%	confirmed
P02	FFPE	51	*PIK3CA*	p.H1047L	NGS	IGV and Pipeline	12	31	39%	confirmed
P26	FFPE	nd	*PIK3CA*	p.E542K	NGS	IGV	0	0		not confirmed
P37	PE	21	*ROS1*	p.D2033N	NGS	IGV and Pipeline	3	3	100%	confirmed
Overview of Additional Variants that were Not Reported by MD										
P03	FFPE	nd	*EGFR*	p.V834L	NGS; FISH	Pipeline	9	64	14%	na
P34_S1	FFPE	76	*KRAS*	p.L56fs	NGS; FISH	Pipeline	7	33	21%	na
P39	FFPE	65	*NRAS*	p.G15fs	NGS; FISH	Pipeline	5	22	23%	na
P40	FFPE	68	*BRAF*	p.Y472fs	NGS; FISH	Pipeline	6	13	46%	na

MD = medical diagnostics; ^a^ read counts for pipeline data if available; ^b^ calculated using average of coverage in *MET* exon 13 and 15 minus the coverage in *MET* exon 14; ^c^ not calculated when total reads is <3; nd = not determined; na = not applicable. FFPE = formalin-fixed paraffin-embedded, PE = pleural effusion.

**Table 2:** Overview of fusion gene/FISH break positive samples analyzed by the all-in-one transcriptome-based assay and the summary of the results.

Sample ID	Origin	DV200	MD Variant			Results of All-In-One Transcriptome-Based Assay				
			Gene	IHC	FISH	Fusion Transcript	IGV (Splitting Reads in Gene 1, Splitting Reads in Gene 2)	Fusion Catcher (Spanning, Splitting Reads)	Strand NGS (Splitting Reads)	Status
H2228	cell line	nd	*ALK*	nd	nd	EML4_E6-ALK_E20	104, 59	41, 107	84	confirmed
P07	FFPE	65	*ALK*	+	+					not confirmed
P08	FFPE	67	*ALK*	+	+	KIF5B_E24-ALK_E20	5, 9			confirmed
P13	PE	89	*ALK*	+	nd	EML4_E6-ALK_E20	83, 238	58, 185	170	confirmed
P14	PE	86	*ALK*	+	nd	DCTN1_E26-ALK_E20	76, 21	20, 41	51	confirmed
P18	Frozen	86	*ALK*	+	+	EML4_E6-ALK_E20	230, 143	74, 290	789	confirmed
P33	FFPE	58	*ALK*	+	+	EML4_E6-ALK_E20	6, 4			confirmed
P34_S1	Frozen	82	*ALK*	+	+	EML4_E6-ALK_E20	62, 41	44, 156	77	confirmed
P34_S1	FFPE	76	*ALK*	+	+	EML4_E6-ALK_E20	7, 3	2, 3	2	confirmed
P34_S2	FFPE	70	*ALK*	+	+	EML4_E6-ALK_E20	38, 17	10, 3	2	confirmed
P36	PE	93	*ALK*	+	+	EML4_E6-ALK_E18	49, 105	35, 156	86	confirmed
P42	PE	53	*ALK*	+	+	MPRIP_E21-ALK_E20	8, 20	5, 26	27	confirmed
KM12	cell line	94	*NTRK1*	nd	nd	TPM3_E7-NTRK1_E9	188, 87	41, 153	340	confirmed
P08	FFPE	67	*RET*	nd	+					true negative
P11	FFPE	81	*RET*	nd	+	KIF5B_E15-RET_E12	2, 3			confirmed
P37	PE	21	*ROS1*	nd	+	CD74_E6-ROS1_E34	0, 3	2, 3	1	confirmed
P38	FFPE	55	*ROS1*	nd	+	EZR_E10-ROS1_E34	11, 2	4, 3		confirmed
P41	FFPE	nd	*ROS1*	nd	+	EZR_E10-ROS1_E34	19, 0	10, 9		confirmed

MD: molecular diagnostics; PE: pleural effusion; FFPE: formalin-fixed paraffin-embedded; nd: not done. For P34_S1, the tissue sample was split into two parts; one part was frozen and the other part was used to generate FFPE material.

**Table 3:** Sensitivity of the assay calculated before and after implementation of the quality criteria.

Variant Type	All Variants (Sensitivity)	Variants in Samples with DV200 >30 and Unique Read Count >50 K (Sensitivity)
SNVs/INDELs	32/39 (82%)	19/19 (100%)
*MET* exon skipping	3/3 (100%)	3/3 (100%)
Fusions	17/18 (94%)	13/13 (100%)
Overall	52/60 (87%)	35/35 (100%)

## Supplementary Materials

The following are available online at https://www.mdpi.com/2072-6694/12/10/2843/s1, Figure S1: IGV screenshot of EGFR exon 19 with some correctly aligned reads with additional soft-clipping reads as a result of exon 19 deletion in P22, Figure S2: Schematic presentation of the final cDNA fragments of the sequence libraries, Table S1: QC data of our all-in-one transcriptome-based NGS assay, Table S2: Overview of RNA-based ddPCR results, Table S3: Overview of NanoString results on samples with expected fusions, Table S4: Progression free survival of ALK positive patients, Table S5: Overview of variant calling for samples sequenced with low and normal library input, Table S6: Overview of variant calling for samples with normal and low RNA input, Table S7: Overview of variant calling results in relation to QC data, Table S8: Linear Regression analysis to identify variables associated with percentage of unique reads, Table S9: Number of landing probes per gene in the three designs, File S1: landingprobes.
