# Supplementary material for: An All-In-One Transcriptome-Based Assay to Identify Therapy-Guiding Genomic Aberrations in Nonsmall Cell Lung Cancer Patients
Source: Cancers (Basel). 2020 Oct 1;12(10):2843. doi: 10.3390/cancers12102843 (PMC7650834; doi:10.3390/cancers12102843)
Supplement: Supplementary file 1 [file cancers-12-02843-s001.zip › cancers-943991 - supplementary material.docx]

Supplementary Materials

An All-In-One Transcriptome-Based Assay to Identify Therapy-Guiding Genomic Aberrations in Nonsmall Cell Lung Cancer Patients

Jiacong Wei, Anna A. Rybczynska, Pei Meng, Martijn Terpstra, Ali Saber, Jantine Sietzema, Wim Timens, Ed Schuuring, T. Jeroen N. Hiltermann,
Harry. J.M. Groen, Anthonie J. van der Wekken, Anke van den Berg and Klaas Kok


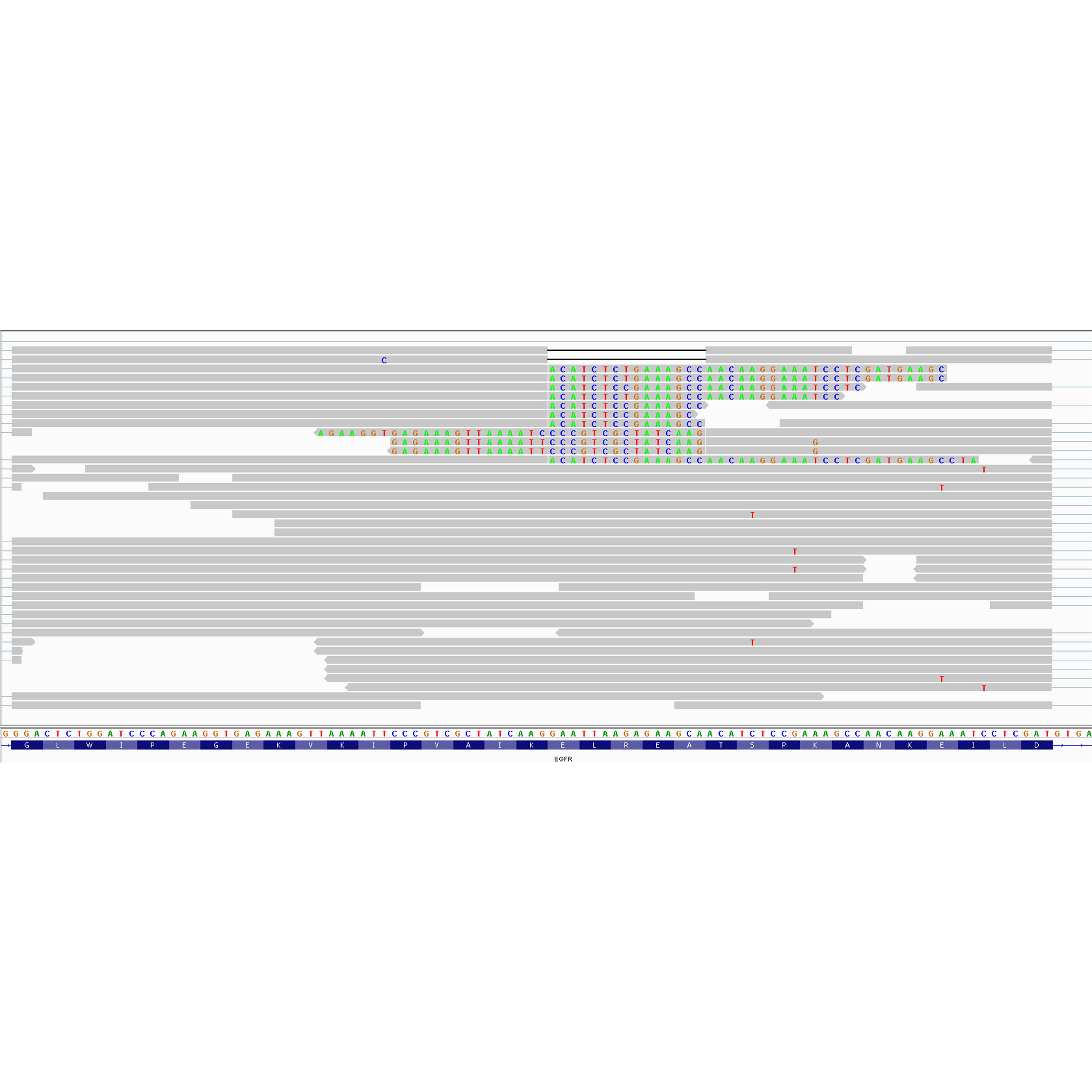


**Figure S1.** IGV screenshot of EGFR exon 19 with some correctly aligned reads with additional soft-clipping reads as a result of exon 19 deletion in P22. The total coverage was 38, with 2 reads with a deletion showing the correct alignment and 11 reads with an exon 19 deletion for which the reads were miss-aligned.


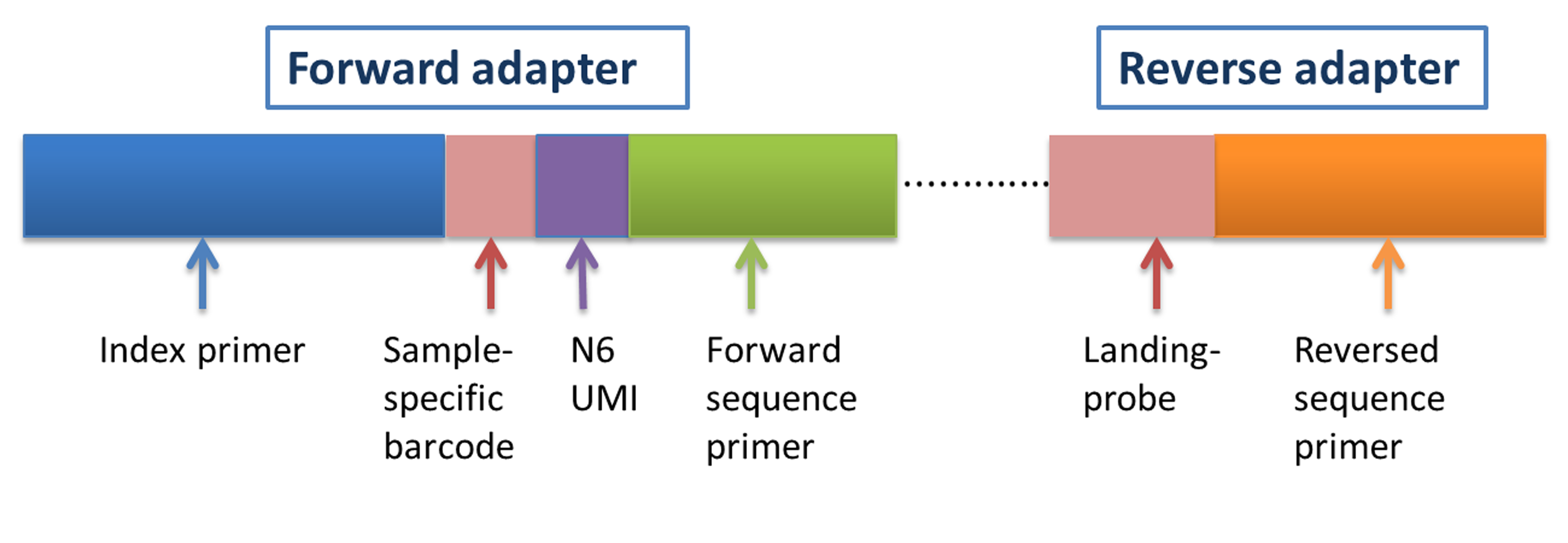


Forward adapter sequence

5’*AATGATACGGCGACCACCGAGATCTACAC*AGAGCACACGTCTGAACTCCAGTCACX_8_N_6_ TCTTTCCCTACACGACGCTCTTCCGATCT(N_40_)

Reversed adapter sequence
5’-(N_40_) AGATCGGAAGAGCACACGTC *ATCTCGTATGCCGTCTTCTGCTTG* 3’

Italic: PCR primers

X_8_: sample specific barcode

N_6_: unique molecular identifier

N_40_: mutation hotspot-specific landing-probe

……..: genomic segment to be sequenced

**Figure S2.** Schematic presentation of the final cDNA fragments of the sequence libraries. Various components of the adapter sequences are indicated, with the full sequence of the adapters given below the figure.

**Table S1.** QC data of our all-in-one transcriptome-based NGS assay.

| **Sample ID** | **Design** | **Origin** | **Year of FFPE** | **RNA Input (ng)** | **DV 200** | **Ct Value** | **Ampl. Cycles** | **Tapestation Average Size (bp)** | **Kapa qPCR on Library Molarity (pmol/l)** | **Panel Version** | **Clean Reads** | **Q20** | **Unique Reads on Target** | **U%** |
| --- | --- | --- | --- | --- | --- | --- | --- | --- | --- | --- | --- | --- | --- | --- |
| A549 | 1 | cell line | na | 200 | nd | 28 | 28 | 508 | nd | D1 | 3,101,968 | 99 | 284,952 | 9% |
| H1299 | 1 | cell line | na | 121 | nd | 25 | 25 | 429 | nd | D1 | 2,927,998 | 99 | 321,756 | 11% |
| H1650 | 1 | cell line | na | 200* | nd | 26 | 27 | 415 | nd | D1 | 1,235,674 | 97 | 97,845 | 8% |
| H1975 | 1 | cell line | na | 200 | nd | 27 | 28 | 508 | nd | D1 | 1,440,484 | 99 | 235,397 | 16% |
| H2228 | 1 | cell line | na | 200* | nd | 21 | 22 | 436 | nd | D1 | 2,640,582 | 99 | 222,701 | 8% |
| H596 | 2 | cell line | na | 200 | 98 | 28 | 29 | 370 | 449 | D2 | 2,548,806 | 98 | 1,262,964 | 50% |
| H820 | 2 | cell line | na | 200 | 99 | 22 | 24 | 451 | 3,699 | D2 | 1,993,700 | 96 | 618,358 | 31% |
| HCT116 | 1 | cell line | na | 188 | nd | 21 | 22 | 487 | nd | D1 | 1,908,568 | 99 | 289,423 | 15% |
| Hs746T | 2 | cell line | na | 200 | 97 | 22 | 24 | 466 | 3,932 | D2 | 1,852,642 | 95 | 639,210 | 35% |
| KM12 | 2 | cell line | na | 200 | 94 | 23 | 24 | 491 | 2,946 | D2 | 2,394,708 | 98 | 792,640 | 33% |
| KOPN-8 | 2 | cell line | na | 200 | 99 | 21 | 24 | 457 | 6,548 | D2 | 1,855,904 | 99 | 201,459 | 11% |
| P01 | 2 | PE | na | 137 | 90 | 25 | 26 | 458 | 1,032 | D2 | 2,502,766 | 98 | 199,647 | 8% |
| P02 | 2 | FFPE | 2015 | 500 | 51 | 27 | 30 | 328 | 3,865 | D2 | 1,747,596 | 98 | 36,593 | 2% |
| P03 | 1 | FFPE | 2015 | 500 | nd | 29 | 32 | 356 | nd | D1 | 3,765,136 | 97 | 67,381 | 2% |
| P04_S1 | 3 | FFPE | 2017 | 500 | 26 | 24 | 25 | 372 | 693 | D3 | 3,193,166 | 98 | 221,944 | 7% |
| P04_S2 | 3 | PE | na | 200 | 88 | 22 | 23 | 459 | 2,976 | D3 | 3,896,308 | 99 | 1,331,315 | 34% |
| P05 | 2 | PE | na | 200 | 17 | 26 | 29 | 364 | 4,450 | D2 | 1,960,866 | 98 | 167,871 | 9% |
| P06 | 1 | FFPE | 2016 | 500 | 37 | 29 | 32 | 338 | nd | D1 | 1,606,174 | 97 | 23,351 | 1% |
| P07 | 2 | FFPE | 2016 | 500 | 65 | 25 | 26 | 379 | 1,270 | D2 | 2,219,310 | 98 | 41,696 | 2% |
| P08 | 1 | FFPE | 2016 | 500 | 67 | 25 | 26 | 359 | nd | D1 | 4,665,964 | 97 | 178,860 | 4% |
| P09 | 3 | FFPE | 2017 | 500 | 38 | 23 | 25 | 324 | 1,104 | D3 | 2,802,770 | 98 | 308,991 | 11% |
| P10 | 3 | FFPE | 2017 | 500 | 36 | 24 | 25 | 349 | 234 | D3 | 2,408,928 | 98 | 76,990 | 3% |
| P11 | 2 | FFPE | 2017 | 500 | 81 | 27 | 29 | 346 | 1,396 | D2 | 2,318,920 | 98 | 79,747 | 3% |
| P13 | 3 | PE | na | 200 | 89 | 21 | 22 | 476 | 2,763 | D3 | 2,605,532 | 98 | 1,297,907 | 50% |
| P14 | 2 | PE | na | 200 | 86 | 22 | 24 | 462 | 2,176 | D2 | 2,197,688 | 98 | 600,434 | 27% |
| P15 | 1 | FFPE | 2014 | 304 | 40 | 28 | 32 | 358 | nd | D1 | 2,827,802 | 97 | 79,865 | 3% |
| P16 | 3 | FFPE | 2017 | 25 | 50 | 24 | 25 | 411 | 87 | D3 | 16,938 | 98 | 1,223 | 7% |
| P17 | 1 | FFPE | 2014 | 195 | 57 | 25 | 25 | 369 | nd | D1 | 315,038 | 97 | 79,759 | 25% |
| P18 | 2 | Frozen | na | 200 | 86 | 23 | 25 | 447 | 2,681 | D2 | 4,500,346 | 98 | 466,598 | 10% |
| P19 | 3 | FFPE | 2017 | 200 | 47 | 24 | 25 | 372 | 309 | D3 | 2,796,480 | 98 | 81,208 | 3% |
| P21 | 2 | FFPE | 2017 | 432 | 34 | 27 | 29 | 337 | 1,403 | D2 | 2,261,980 | 98 | 80,234 | 4% |
| P22 | 2 | FFPE | 2014 | 500 | 69 | 24 | 26 | 353 | 2,648 | D2 | 1,638,530 | 98 | 86,309 | 5% |
| P23 | 2 | FFPE | 2015 | 500 | 44 | 28 | 30 | 343 | 478 | D2 | 2,544,046 | 98 | 19,638 | 1% |
| P25 | 1 | FFPE | 2015 | 500 | 71 | 26 | 29 | 364 | nd | D1 | 3,346,274 | 96 | 139,373 | 4% |
| P26 | 1 | FFPE | 2014 | 500 | nd | 29 | 32 | 341 | nd | D1 | 3,134,408 | 96 | 59,613 | 2% |
| P28 | 3 | FFPE | 2017 | 380 | 38 | 24 | 25 | 344 | 309 | D3 | 1,990,944 | 98 | 88,560 | 4% |
| P29 | 1 | FFPE | 2014 | 500 | nd | 29 | 32 | 343 | nd | D1 | 2,811,104 | 97 | 75,307 | 3% |
| P31 | 1 | FFPE | 2014 | 500 | 66 | 25 | 28 | 356 | nd | D1 | 2,009,752 | 97 | 211,390 | 11% |
| P32 | 2 | FFPE | 2011 | 500 | 32 | 28 | 29 | 394 | 135 | D2 | 812,936 | 99 | 4,915 | 1% |
| P33 | 2 | FFPE | 2012 | 500 | 58 | 28 | 30 | 361 | 990 | D2 | 1,856,548 | 98 | 21,902 | 1% |
| P34_S1 | 2 | FFPE | 2013 | 500 | 76 | 25 | 27 | 351 | 2,729 | D2 | 2,117,588 | 98 | 78,515 | 4% |
| P34_S1 | 1 | Frozen | na | 160 | 82 | 20 | 21 | 489 | nd | D1 | 5,066,278 | 96 | 455,287 | 9% |
| P34_S2 | 2 | FFPE | 2013 | 500 | 70 | 24 | 25 | 350 | 2,633 | D2 | 2,347,452 | 97 | 145,018 | 6% |
| P35 | 3 | PE | na | 200 | 81 | 21 | 22 | 425 | 3,333 | D3 | 3,312,072 | 97 | 1,276,594 | 39% |
| P36 | 2 | PE | na | 200 | 93 | 21 | 22 | 461 | 3,320 | D2 | 2,353,046 | 98 | 1,037,592 | 44% |
| P37 | 2 | PE | na | 200 | 21 | 25 | 26 | 432 | 1,002 | D2 | 2,407,286 | 97 | 232,418 | 10% |
| P38 | 2 | FFPE | 2014 | 500 | 55 | 24 | 26 | 340 | 4,444 | D2 | 1,873,690 | 98 | 107,167 | 6% |
| P39 | 2 | FFPE | 2014 | 500 | 65 | 27 | 28 | 359 | 1,551 | D2 | 1,847,672 | 98 | 57,088 | 3% |
| P40 | 2 | FFPE | 2014 | 500 | 68 | 27 | 28 | 376 | 571 | D2 | 2,233,814 | 98 | 42,301 | 2% |
| P41 | 1 | FFPE | 2015 | 380 | nd | 27 | 30 | 333 | nd | D1 | 1,617,424 | 97 | 67,491 | 4% |
| P42 | 2 | PE | na | 200 | 53 | 24 | 26 | 401 | 2,352 | D2 | 2,769,332 | 98 | 599,272 | 22% |
| Control Samples | | | | | | | | | | | | | | |
| KM12^a^ | 2 | cell line | na | 50 | 96 | 24 | 26 | 487 | 1,978 | D2 | 2,155,384 | 96 | 253,719 | 12% |
| KM12^a^ | 2 | cell line | na | 10 | 96 | 26 | 28 | 460 | 2,625 | D2 | 2,329,356 | 96 | 148,920 | 6% |
| P34_S1^a^ | 2 | Frozen | na | 50 | 80 | 23 | 24 | 436 | 3,338 | D2 | 1,925,044 | 96 | 482,731 | 25% |
| P34_S1^a^ | 2 | Frozen | na | 10 | 81 | 25 | 28 | 426 | 6,751 | D2 | 1,979,056 | 96 | 188,064 | 10% |
| P42^a^ | 2 | PE | na | 50 | 51 | 26 | 28 | 404 | 2,814 | D2 | 1,952,360 | 96 | 139,801 | 7% |
| P42^a^ | 2 | PE | na | 10 | 45 | 27 | 28 | 462 | 474 | D2 | 2,052,066 | 96 | 49,665 | 2% |
| P31^b^ | 2 | FFPE | 2014 | 500 | 66 | 26 | 28 | 387 | 1,229 | D2 | 2,120,348 | 98 | 66,964 | 3% |
| P15^b^ | 2 | FFPE | 2014 | 500 | 43 | 25 | 27 | 372 | 1,051 | D2 | 1,731,948 | 98 | 73,397 | 4% |
| P34_S1^b^ | 2 | Frozen | na | 200 | 82 | 26 | 28 | 407 | 2,611 | D2 | 1,697,218 | 98 | 90,148 | 5% |
| P25^C^ | 1 | FFPE | 2015 | na | na | na | na | na | na | D1 | 532,946 | 98 | 86,792 | 16% |
| P03^C^ | 1 | FFPE | 2015 | na | na | na | na | na | na | D1 | 332,448 | 98 | 22,639 | 7% |
| P17^C^ | 1 | FFPE | 2014 | na | na | na | na | na | na | D1 | 315,038 | 98 | 79,759 | 25% |
| P29^C^ | 1 | FFPE | 2014 | na | na | na | na | na | na | D1 | 252,738 | 98 | 26,561 | 11% |
| P15^C^ | 1 | FFPE | 2014 | na | na | na | na | na | na | D1 | 250,314 | 98 | 29,124 | 12% |
| P34_S1^C^ | 1 | Frozen | na | na | na | na | na | na | na | D1 | 274,416 | 98 | 57,478 | 21% |

* samples of which we lost 1/3 to 1/4 of the RNA accidently during library preparation that were excluded from linear regression analysis by SPSS, control samples were not analyzed in linear regression analysis by SPSS; ^a^ samples analyzed with lower input; ^b^ samples analyzed both in design one and design two; ^c^ samples of which 1/8 of the required library input was pooled and sequenced; U%: percentage of unique reads; PE: pleural effusion; FFPE: formalin-fixed paraffin-embedded tissue; na: not applicable; nd: not done.

**Table S2.** Overview of RNA-based ddPCR results.

| **Sample ID** | **Origin** | **DV 200** | **MD Variant** | | **NGS Result** | | | **ddPCR Validation** | | | | |  |
| --- | --- | --- | --- | --- | --- | --- | --- | --- | --- | --- | --- | --- | --- |
|  |  |  | **Gene** | **Amino Acid Change** | **Mutant Reads** | **Total Reads** | **VAF** | **Input RNA (ng)** | **Mutant Copies** | **Wild Type Copies** | **Fractional Abundance** | **Wells** |  |
| Variants Detected by Our All-In-One Assay | | | | | | | | | | | | | |
| A549 | cell line | na | *KRAS* | p.G12S | 512 | 513 | 100% | 1 | 380 | 0 | 100% | 2 |  |
| H1650 | cell line | na | *EGFR* | E19 DEL | 72 | 98 | 73% | 1 | 2350 | 1110 | 68% | 2 |  |
| H1975 | cell line | na | *EGFR* | p.L858R | 564 | 684 | 82% | 1 | 1988 | 402 | 83% | 2 |  |
| H1975 | cell line | na | *EGFR* | p.T790M | 347 | 425 | 82% | 2 | 4831 | 996 | 83% | 1 |  |
| HCT116 | cell line | na | *KRAS* | p.G13D | 223 | 456 | 49% | 1 | 259 | 217 | 54% | 2 |  |
| KOPN-8 | cell line | 99 | *KRAS* | p.G12D | 99 | 177 | 56% | 1 | 268 | 283 | 49% | 2 |  |
| P01 | PE | 90 | *KRAS* | p.G12D | 14 | 111 | 13% | 8 | 64 | 364 | 15% | 1 |  |
| P03 | FFPE | na | *KRAS* | p.G12A | 8 | 8 | 100% | 8 | 10 | 0 | 100% | 1 |  |
| P06 | FFPE | 37 | *EGFR* | E19 DEL | 20 | 28 | 71% | 5 | 527 | 55 | 91% | 3 |  |
| P15 | FFPE | 40 | *EGFR* | E19 DEL | 119 | 143 | 83% | 5 | 629 | 165 | 79% | 3 |  |
| P17 | FFPE | 57 | *EGFR* | E19 DEL* | 311 | 373 | 83% | 8 | 1540 | 203 | 88% | 2 |  |
| P17 | FFPE | 57 | *EGFR* | p.T790M | 62 | 127 | 49% | 6 | 1420 | 614 | 70% | 1 |  |
| P22 | FFPE | 69 | *EGFR* | E19 deletion | 13 | 38 | 34% | 8 | 186 | 167 | 53% | 2 |  |
| P39 | FFPE | 65 | *KRAS* | p.G12D | 8 | 12 | 67% | 8 | 25 | 23 | 53% | 1 |  |
| Variants Not Detected by Our All-In-One Assay | | | | | | | | | | | | | |
| P05 | PE | 17 | *EGFR* | E19 DEL | 0 | 0 | na | 100 | 12 | 9 | 57% | 4 |  |
| P06 | FFPE | 37 | *EGFR* | p.T790M | 0 | 0 | na | 324 | 21 | 188 | 10% | 3 |  |
| P23 | FFPE | 44 | *KRAS* | p.G12C | 0 | 0 | na | 25 | 19 | 8 | 71% | 2 |  |
| P26 | FFPE | na | *EGFR* | p.L858R | 0 | 0 | na | 370 | 177 | 422 | 30% | 4 |  |
| P32 | FFPE | 32 | *KRAS* | p.G12D | 0 | 0 | na | 497 | 0 | 8 | 0% | 3 |  |
| P40 | FFPE | 68 | *KRAS* | p.G12F | 0 | 0 | na | 30 | 95 | 68 | 59% | 1 |  |

* Read counts for EGFR exon 19 are based on IGV; MD: molecular diagnostic; VAF: Variant allele frequency; PE: pleural effusion; FFPE: formalin-fixed paraffin-embedded tissue; na: not analyzed; E19 DEL: exon 19 deletion**.**

**Table S3.** Overview of NanoString results on samples with expected fusions.

| **Sample ID** | **Origin** | **MD*** | **Fusion Transcript  by RNA-Based Assay** | **Nanostring  Validation** |
| --- | --- | --- | --- | --- |
|  |  |  |  |  |
| P07 | FFPE | *ALK* | not detected | Positive |
| P08 | FFPE | *ALK* | KIF5B_E24-ALK_E20 | Positive |
| P08 | FFPE | *RET* | not detected | Negative |
| P11 | FFPE | *RET* | KIF5B_E15-RET_E12 | Positive |
| P18 | FFPE | *ALK* | EML4_E6-ALK_E20 | Positive |
| P34_S1 | FFPE | *ALK* | EML4_E6-ALK_E20 | Positive |
| P38 | FFPE | *ROS1* | EZR_E10-ROS1_E34 | Positive |

* FISH break and/or IHC positive cases as reported by the molecular diagnostics.

**Table S4.** Progression free survival of ALK positive patients.

| **Patient ID** | **FISH** | **IHC** | **Fusion Transcript** | **TKI** | **PFS** |
| --- | --- | --- | --- | --- | --- |
| P07 | + | + | EML4_E6-ALK_E20* | crizotinib | 14 |
| P08 | + | + | KIF5B_E24-ALK_E20 | ceritinib | 19 |
| P13 | + | nd | EML4_E6-ALK_E20 | crizotinib | 8 |
| P14 | + | nd | DCTN1_E26-ALK_E20 | crizotinib /alectinib | no response |
| P18 | + | + | EML4_E6-ALK_E20 | crizotinib | 9 |
| P33 | + | + | EML4_E6-ALK_E20 | crizotinib | 6 |
| P34_S1 | + | + | EML4_E6-ALK_E20 | crizotinib | 15 |
| P34_S2 | + | + | EML4_E6-ALK_E20 | ceritinib | 12 |
| P36 | + | + | EML4_E6-ALK_E18 | crizotinib | 24 |
| P42 | + | + | MPRIP_E21-ALK_E20 | crizotinib | 8 |

* fusion gene detected by NanoString; PFS: progression free survival; TKI: Tyrosine Kinase Inhibitor; nd: not done

**Table S5.** Overview of variant calling for samples sequenced with low and normal library input.

| **Sample ID** | **Gene** | **Variant** | **Standard Library Input** | | | **1/8 Library Input** | | |
| --- | --- | --- | --- | --- | --- | --- | --- | --- |
|  |  |  | **Altered Reads/ Splitting Reads** | **Total Reads/ Spanning Reads** | **MAF** | **Altered Reads/ Splitting Reads** | **Total Reads/ Spanning Reads** | **MAF** |
| P03 | *KRAS* | p.G12A | 8 | 8 | 100% | 4 | 4 | 100% |
| P15 | *EGFR* | E19 deletion | 81 | 110 | 74% | 49 | 58 | 84% |
| P15 | *EGFR* | p.T790M | 22 | 88 | 25% | 13 | 50 | 26% |
| P17 | *EGFR* | E19 deletion | 123 | 143 | 86% | 175 | 198 | 88% |
| P17 | *EGFR* | p.T790M | 62 | 127 | 49% | 92 | 141 | 65% |
| P25 | *BRAF* | p.V600E | 33 | 46 | 72% | 9 | 18 | 50% |
| P34_S1 | *ALK* | fusion | 62 | 41 |  | 7 | 3 |  |

**Table S6.** Overview of variant calling for samples with normal and low RNA input.

| **Sample ID** | **Gene** | **Input** | **Fusion (5’ gene-3’ gene)** | **5' Gene Count-3' Gene Count** |
| --- | --- | --- | --- | --- |
| KM12 | *NTRK1* | 200 | TPM3_E7-NTRK1_E9 | 188, 87 |
| KM12 | *NTRK1* | 50 | TPM3_E7-NTRK1_E9 | 132, 22 |
| KM12 | *NTRK1* | 10 | TPM3_E7-NTRK1_E9 | 88, 26 |
| P34_S1 | *ALK* | 200 | EML4_E6-ALK_E20 | 6, 2 |
| P34_S1 | *ALK* | 50 | EML4_E6-ALK_E20 | 16, 23 |
| P34_S1 | *ALK* | 10 | EML4_E6-ALK_E20 | 9, 10 |
| P42 | *ALK* | 200 | MRPIP_E21-ALK_E20 | 8, 20 |
| P42 | *ALK* | 50 | MRPIP_E21-ALK_E20 | 1, 6 |
| P42 | *ALK* | 10 | MRPIP_E21-ALK_E20 | 0, 6 |

**Table S7.** Overview of variant calling results in relation to QC data.

| **Sample ID** | **Variant 1 (*)** | **Variant 2** | **Variant 3** | **Unique reads** | **RNA input (ng)** | **Origin** | **Year of FFPE** | **DV200** | **Pass QC **** |
| --- | --- | --- | --- | --- | --- | --- | --- | --- | --- |
| A549 | *KRAS* G12S:+ |  |  | 284,952 | 200 | cell line | na | nd | na |
| H1299 | *NRAS* Q61K:+ |  |  | 321,756 | 121 | cell line | na | nd | na |
| H1650 | *EGFR* E746_A750del:+ |  |  | 97,845 | 200 | cell line | na | nd | na |
| H1975 | *EGFR* L858R :+ | *EGFR* T790M :+ |  | 235,397 | 200 | cell line | na | nd | na |
| H820 | *EGFR* E746_A750del:+ | *EGFR* T790M :+ |  | 618,358 | 200 | cell line | na | 99 | Yes |
| KOPN-8 | *KRAS* G12D:+ |  |  | 201,459 | 200 | cell line | na | 99 | Yes |
| HCT116 | *KRAS* G13D:+ | *PIK3CA* H1047R:+ |  | 289,423 | 188 | cell line | na | nd | na |
| H596 | *PIK3CA* E545K:+ | *MET* E14 skipping:+ |  | 1,262,964 | 200 | cell line | na | 98 | Yes |
| Hs746T | *MET* E14 skipping:+ |  |  | 639,210 | 200 | cell line | na | 97 | Yes |
| H2228 | *ALK* fusion:+ |  |  | 222,701 | 200 | cell line | na | nd | na |
| KM12 | *NTRK1* fusion:+ |  |  | 792,640 | 200 | cell line | na | 94 | Yes |
| P18 | *ALK* fusion:+ |  |  | 466,598 | 200 | Frozen | na | 86 | Yes |
| P34_S1 | *ALK* fusion:+ |  |  | 455,287 | 160 | Frozen | na | 82 | Yes |
| P35 | *AKT1* E17K:+ | *BRAF* V600E :+ |  | 1,276,594 | 200 | PE | na | 81 | Yes |
| P05 | *EGFR* E746_A750del:- |  |  | 167,871 | 200 | PE | na | 17 | No |
| P04_S2 | *EGFR* L858R:+ |  |  | 1,331,315 | 200 | PE | na | 88 | Yes |
| P01 | *KRAS* G12D:+ |  |  | 199,647 | 137 | PE | na | 90 | Yes |
| P14 | *ALK* fusion:+ |  |  | 600,434 | 200 | PE | na | 86 | Yes |
| P36 | *ALK* fusion:+ |  |  | 1,037,592 | 200 | PE | na | 93 | Yes |
| P37 | *ROS1* D2033N:+ | *ROS1* fusion:+ |  | 232,418 | 200 | PE | na | 21 | No |
| P42 | *ALK* fusion:+ |  |  | 599,272 | 200 | PE | na | 53 | Yes |
| P13 | *ALK* I1171N:+ | *ALK* G1269A :+ | *ALK* fusion:+ | 1,297,907 | 200 | PE | na | 89 | Yes |
| P25 | *BRAF* V600E:+ |  |  | 139,373 | 500 | FFPE | 2015 | 71 | Yes |
| P15 | *EGFR* E746_A750del:+ | *EGFR* T790M:+ |  | 79,865 | 304 | FFPE | 2014 | 40 | Yes |
| P17 | *EGFR* E746_A750del:+ | *EGFR* T790M:+ |  | 79,759 | 195 | FFPE | 2014 | 57 | Yes |
| P22 | *EGFR* E746_A750del:+ |  |  | 86,309 | 500 | FFPE | 2014 | 69 | Yes |
| P06 | *EGFR* L747_P753delinsS:+ | *EGFR* T790M:- |  | 23,351 | 500 | FFPE | 2016 | 37 | No |
| P26 | *EGFR* L858R:- | *PIK3CA* E542K:- |  | 59,613 | 500 | FFPE | 2014 | nd | na |
| P04_S1 | *EGFR* L858R:+ |  |  | 221,944 | 500 | FFPE | 2017 | 26 | No |
| P03 | *KRAS* G12A:+ |  |  | 67,381 | 500 | FFPE | 2015 | nd | na |
| P28 | *KRAS* G12A:+ |  |  | 88,560 | 380 | FFPE | 2017 | 38 | Yes |
| P23 | *KRAS* G12C:- |  |  | 19,638 | 500 | FFPE | 2015 | 44 | No |
| P32 | *KRAS* G12D:- |  |  | 4,915 | 500 | FFPE | 2011 | 32 | No |
| P39 | *KRAS* G12D:+ |  |  | 57,088 | 500 | FFPE | 2014 | 65 | Yes |
| P40 | *KRAS* G12F:- |  |  | 42,301 | 500 | FFPE | 2014 | 68 | No |
| P31 | *KRAS* Q61H:+ |  |  | 211,390 | 500 | FFPE | 2014 | 66 | Yes |
| P02 | *PIK3CA* H1047L:+ |  |  | 36,593 | 500 | FFPE | 2015 | 51 | No |
| P21 | *MET* E14 skipping:+ |  |  | 80,234 | 432 | FFPE | 2017 | 34 | Yes |
| P07 | *ALK* L1196M:+ | *ALK* Fusion: - |  | 41,696 | 500 | FFPE | 2016 | 65 | No |
| P08 | *ALK* fusion:+ | (*RET* fusion) |  | 178,860 | 500 | FFPE | 2016 | 67 | Yes |
| P33 | *ALK* fusion:+ |  |  | 21,902 | 500 | FFPE | 2012 | 58 | No |
| P34_S1 | *ALK* fusion:+ |  |  | 78,515 | 500 | FFPE | 2013 | 76 | Yes |
| P34_S2 | *ALK* fusion:+ |  |  | 145,018 | 500 | FFPE | 2013 | 70 | Yes |
| P11 | *RET* fusion:+ |  |  | 79,747 | 500 | FFPE | 2017 | 81 | Yes |
| P38 | *ROS1* fusion:+ |  |  | 107,167 | 500 | FFPE | 2014 | 55 | yes |
| P41 | *ROS1* fusion:+ |  |  | 67,491 | 380 | FFPE | 2015 | nd | na |

* variants observed are indicated in black and marked with +; variants not observed are indicated in red and marked with - ; **Pass QC: passed assay quality control; Variants between brackets are true negatives. PE: pleural effusion; FFPE: formalin-fixed paraffin-embedded; na: not applicable; nd: not determined**.**

**Table S8.** Linear Regression analysis to identify variables associated with percentage of unique reads.

| **Variable** | **Univariable Analysis** | | **Multivariable Analysis** | |
| --- | --- | --- | --- | --- |
|  | **ß (95% Confidence)** | ***p* Value** | **ß (95% Confidence)** | ***p* Value** |
| Design Version | 0.063 (0.003, 0.123) | 0.039 | 0.037 (−0.022, 0.096) | 0.215 |
| Material Type | 0.213 (0.15, 0.275) | 0.000 | 0.133 (0.016, 0.249) | 0.027 |
| RNA Input | −0.001 (−0.001, 0) | 0.000 | −0.0001 (−0.0004, 0.0003) | 0.604 |
| DV200 | 0.004 (0.003, 0.006) | 0.000 | 0.002 (0, 0.004) | 0.019 |
| PCR Cycles | −0.03 (−0.041, −0.019) | 0.000 | −0.006 (−0.023, 0.011) | 0.488 |

**Table S9.** Number of landing probes per gene in the three designs.

| **Category** | **Gene** | **Landing Probe Count** | | |
| --- | --- | --- | --- | --- |
|  |  | **Design 1** | **Design 2** | **Design 3** |
| mutation | *AKT1* |  | 2 | 1 |
| mutation | *BRAF* | 7 | 4 | 7 |
| mutation | *DDR2* |  | 5 | 2 |
| mutation | *EGFR* | 10 | 11 | 10 |
| mutation | *ERBB2* |  | 5 | 5 |
| mutation | *HRAS* |  | 6 | 7 |
| mutation | *JAK2* |  | 2 | 17 |
| mutation | *KIT* |  | 15 | 14 |
| mutation | *KRAS* | 4 | 6 | 7 |
| mutation | *MAP2K1* |  | 13 | 13 |
| mutation | *NRAS* | 4 | 11 | 8 |
| mutation | *PDGFRA* |  | 8 | 8 |
| mutation | *PIK3CA* | 6 | 8 | 9 |
| mutation | *POLE* |  | 5 | 12 |
| mutation | *STK11* | 5 |  |  |
| mutation | *STAT3* |  |  | 12 |
| mutation | *TP53* |  |  | 27 |
| exon skipping | *MET* | 111 | 14 | 11 |
| fusion | *ALK* | 70 | 105 | 70 |
| fusion | *RET* | 60 | 98 | 58 |
| fusion | *ROS1* | 92 | 140 | 140 |
| fusion | *KMT2A* |  | 14 |  |
| fusion | *MLLT1* |  | 14 |  |
| fusion | *NRG1* |  |  | 34 |
| fusion | *NTRK1* |  | 20 | 46 |
| fusion partner | *ATP1B1* |  |  | 34 |
| fusion partner | *CCDC6* | 48 | 86 | 24 |
| fusion partner | *CD74* | 24 | 34 | 26 |
| fusion partner | *EML4* | 62 | 86 | 50 |
| fusion partner | *EZR* | 36 | 54 | 38 |
| fusion partner | *HIP1* | 87 | 116 | 66 |
| fusion partner | *KIF5B* | 70 | 100 | 62 |
| fusion partner | *SDC4* | 22 | 38 | 12 |
| fusion partner | *SLC34A2* | 44 | 65 | 32 |
| expression | *BRCA1* |  |  | 8 |
| expression | *BRCA2* |  |  | 8 |
| expression | *HPRT1* |  | 6 |  |
| expression | *MPRIP* |  | 10 | 10 |
| expression | *MYC* | 18 | 4 | 4 |
| expression | *MYCN* | 21 | 4 |  |
| expression | *POLR2A* |  | 4 | 12 |
| expression | *PXDNL* |  |  | 8 |
| expression | *RYR1* |  |  | 8 |
| expression | *SCN8A* |  |  | 6 |
| expression | *SLIT3* |  |  | 12 |
| expression | *FGFR1* | 60 | 6 | 4 |
| expression | *FGFR3* | 47 | 6 | 6 |
| expression | *RICTOR* |  |  | 12 |
| gender control | *AMELY* |  | 2 | 2 |
| housekeeping | *B2M* |  |  | 14 |
| housekeeping | *CD274* |  |  | 8 |
| housekeeping | *GAPDH* | 8 |  |  |
| housekeeping | *HRPT1* | 59 |  |  |
| housekeeping | *GP9* |  | 4 | 4 |
| housekeeping | *GUSB* |  |  | 9 |
| housekeeping | *HAVCR2* |  |  | 6 |
| housekeeping | *HMBS* |  |  | 4 |
| housekeeping | *IPO8* |  | 4 | 6 |
| housekeeping | *PAPPA2* |  |  | 22 |
| housekeeping | *PPIA* |  |  | 4 |
| housekeeping | *PTPRC* |  | 8 | 6 |
| housekeeping | *RP2* | 42 | 6 | 6 |
| housekeeping | *RPS9* |  |  | 8 |
| housekeeping | *TBP* |  |  | 11 |
| housekeeping | *UBC* |  |  | 2 |
| housekeeping | *YWHAZ* |  |  | 10 |

| 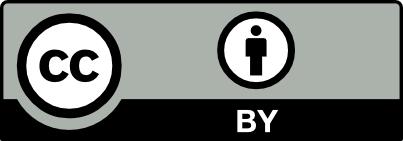 | © 2020 by the authors. Licensee MDPI, Basel, Switzerland. This article is an open access article distributed under the terms and conditions of the Creative Commons Attribution (CC BY) license (http://creativecommons.org/licenses/by/4.0/). |
| --- | --- |
