# Supplementary material for: An All-In-One Transcriptome-Based Assay to Identify Therapy-Guiding Genomic Aberrations in Nonsmall Cell Lung Cancer Patients
Source: Cancers (Basel). 2020 Oct 1;12(10):2843. doi: 10.3390/cancers12102843 (PMC7650834; doi:10.3390/cancers12102843)
Supplement: Supplementary file 1 [file cancers-12-02843-s001.zip › cancers-943991 - supplementary-/cancers-943991 - supplementary material.pdf]

Supplementary Materials

An All-In-One Transcriptome-Based Assay to Identify Therapy-Guiding Genomic Aberrations in Nonsmall Cell Lung Cancer Patients

Jiacong Wei, Anna A. Rybczynska, Pei Meng, Martijn Terpstra, Ali Saber, Jantine Sietzema, Wim Timens, Ed Schuuring, T. Jeroen N. Hiltermann, Harry. J.M. Groen, Anthonie J. van der Wekken, Anke van den Berg and Klaas Kok

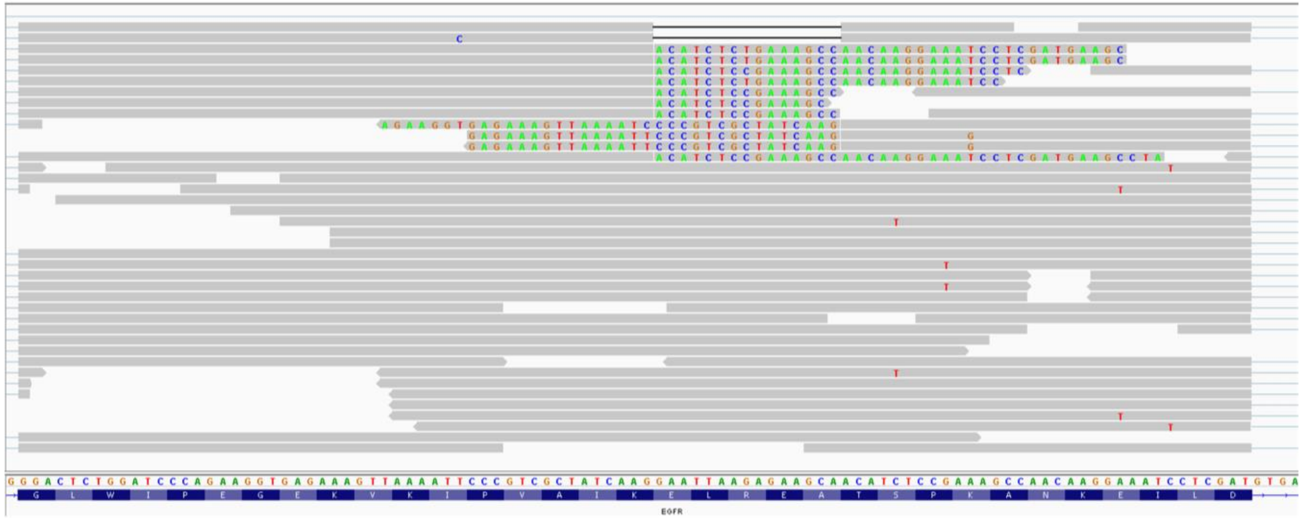

Figure S1. IGV screenshot of EGFR exon 19 with some correctly aligned reads with additional soft-clipping reads as a result of exon 19 deletion in P22. The total coverage was 38, with 2 reads with a deletion showing the correct alignment and 11 reads with an exon 19 deletion for which the reads were miss-aligned.

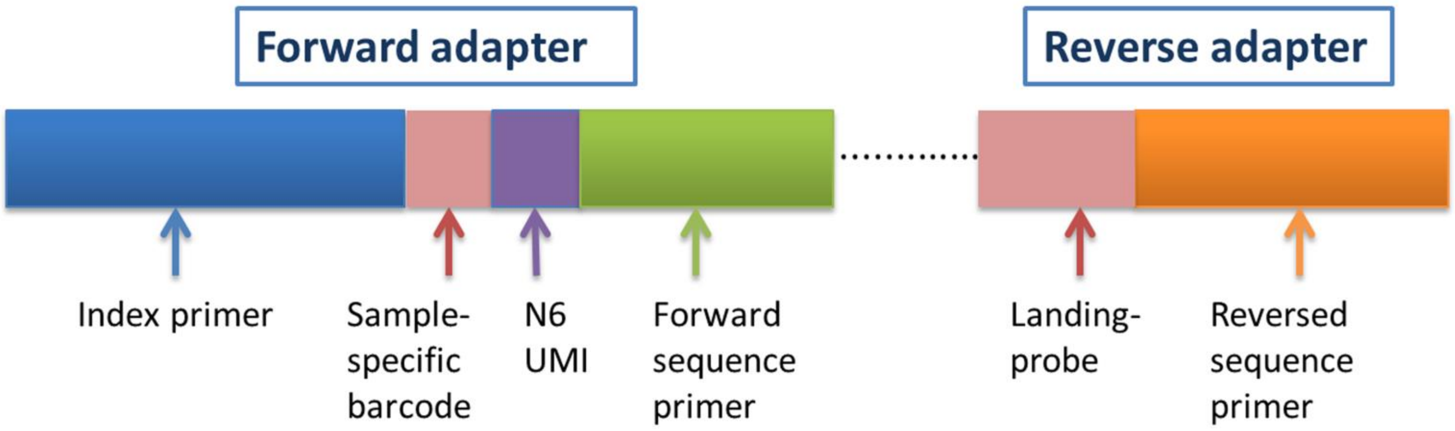

Forward adapter sequence
5′*AATGATACGGCGACCACCGAGATCTACAC*AGAGCACACGTCTGAACTCCAGTCACX_8N_6 TCTTTCCCTACACGACGCTCTTCCGATCT(N_{40})
Reversed adapter sequence
5′-(N_{40}) AGATCGGAAGAGCACACGTC *ATCTCGTATGCCGTCTTCTGCTTG* 3′
Italic: PCR primers
X_8: sample specific barcode
N_6: unique molecular identifier
N_{40}: mutation hotspot-specific landing-probe
……..: genomic segment to be sequenced

Figure S2. Schematic presentation of the final cDNA fragments of the sequence libraries. Various components of the adapter sequences are indicated, with the full sequence of the adapters given below the figure.

Table S1. QC data of our all-in-one transcriptome-based NGS assay.

Sample ID	Design	Origin	Year of FFPE	RNA Input (ng)	DV 200	Ct Value	Ampl. Cycles	Tapestation Average Size (bp)	Kapa qPCR on Library Molarity (pmol/l)	Panel Version	Clean Reads	Q20	Unique Reads on Target	U%
A549	1	cell line	na	200	nd	28	28	508	nd	D1	3,101,968	99	284,952	9%
H1299	1	cell line	na	121	nd	25	25	429	nd	D1	2,927,998	99	321,756	11%
H1650	1	cell line	na	200*	nd	26	27	415	nd	D1	1,235,674	97	97,845	8%
H1975	1	cell line	na	200	nd	27	28	508	nd	D1	1,440,484	99	235,397	16%
H2228	1	cell line	na	200*	nd	21	22	436	nd	D1	2,640,582	99	222,701	8%
H596	2	cell line	na	200	98	28	29	370	449	D2	2,548,806	98	1,262,964	50%
H820	2	cell line	na	200	99	22	24	451	3,699	D2	1,993,700	96	618,358	31%
HCT116	1	cell line	na	188	nd	21	22	487	nd	D1	1,908,568	99	289,423	15%
Hs746T	2	cell line	na	200	97	22	24	466	3,932	D2	1,852,642	95	639,210	35%
KM12	2	cell line	na	200	94	23	24	491	2,946	D2	2,394,708	98	792,640	33%
KOPN-8	2	cell line	na	200	99	21	24	457	6,548	D2	1,855,904	99	201,459	11%
P01	2	PE	na	137	90	25	26	458	1,032	D2	2,502,766	98	199,647	8%
P02	2	FFPE	2015	500	51	27	30	328	3,865	D2	1,747,596	98	36,593	2%
P03	1	FFPE	2015	500	nd	29	32	356	nd	D1	3,765,136	97	67,381	2%
P04_S1	3	FFPE	2017	500	26	24	25	372	693	D3	3,193,166	98	221,944	7%
P04_S2	3	PE	na	200	88	22	23	459	2,976	D3	3,896,308	99	1,331,315	34%
P05	2	PE	na	200	17	26	29	364	4,450	D2	1,960,866	98	167,871	9%
P06	1	FFPE	2016	500	37	29	32	338	nd	D1	1,606,174	97	23,351	1%
P07	2	FFPE	2016	500	65	25	26	379	1,270	D2	2,219,310	98	41,696	2%
P08	1	FFPE	2016	500	67	25	26	359	nd	D1	4,665,964	97	178,860	4%
P09	3	FFPE	2017	500	38	23	25	324	1,104	D3	2,802,770	98	308,991	11%
P10	3	FFPE	2017	500	36	24	25	349	234	D3	2,408,928	98	76,990	3%
P11	2	FFPE	2017	500	81	27	29	346	1,396	D2	2,318,920	98	79,747	3%
P13	3	PE	na	200	89	21	22	476	2,763	D3	2,605,532	98	1,297,907	50%
P14	2	PE	na	200	86	22	24	462	2,176	D2	2,197,688	98	600,434	27%
P15	1	FFPE	2014	304	40	28	32	358	nd	D1	2,827,802	97	79,865	3%
P16	3	FFPE	2017	25	50	24	25	411	87	D3	16,938	98	1,223	7%
P17	1	FFPE	2014	195	57	25	25	369	nd	D1	315,038	97	79,759	25%
P18	2	Frozen	na	200	86	23	25	447	2,681	D2	4,500,346	98	466,598	10%
P19	3	FFPE	2017	200	47	24	25	372	309	D3	2,796,480	98	81,208	3%
P21	2	FFPE	2017	432	34	27	29	337	1,403	D2	2,261,980	98	80,234	4%
P22	2	FFPE	2014	500	69	24	26	353	2,648	D2	1,638,530	98	86,309	5%
P23	2	FFPE	2015	500	44	28	30	343	478	D2	2,544,046	98	19,638	1%
P25	1	FFPE	2015	500	71	26	29	364	nd	D1	3,346,274	96	139,373	4%
P26	1	FFPE	2014	500	nd	29	32	341	nd	D1	3,134,408	96	59,613	2%
P28	3	FFPE	2017	380	38	24	25	344	309	D3	1,990,944	98	88,560	4%
P29	1	FFPE	2014	500	nd	29	32	343	nd	D1	2,811,104	97	75,307	3%

Sample ID	Design	Origin	Year of FFPE	RNA Input (ng)	DV 200	Ct Value	Ampl. Cycles	Tapestation Average Size (bp)	Kapa qPCR on Library Molarity (pmol/l)	Panel Version	Clean Reads	Q20	Unique Reads on Target	U%
P31	1	FFPE	2014	500	66	25	28	356	nd	D1	2,009,752	97	211,390	11%
P32	2	FFPE	2011	500	32	28	29	394	135	D2	812,936	99	4,915	1%
P33	2	FFPE	2012	500	58	28	30	361	990	D2	1,856,548	98	21,902	1%
P34_S1	2	FFPE	2013	500	76	25	27	351	2,729	D2	2,117,588	98	78,515	4%
P34_S1	1	Frozen	na	160	82	20	21	489	nd	D1	5,066,278	96	455,287	9%
P34_S2	2	FFPE	2013	500	70	24	25	350	2,633	D2	2,347,452	97	145,018	6%
P35	3	PE	na	200	81	21	22	425	3,333	D3	3,312,072	97	1,276,594	39%
P36	2	PE	na	200	93	21	22	461	3,320	D2	2,353,046	98	1,037,592	44%
P37	2	PE	na	200	21	25	26	432	1,002	D2	2,407,286	97	232,418	10%
P38	2	FFPE	2014	500	55	24	26	340	4,444	D2	1,873,690	98	107,167	6%
P39	2	FFPE	2014	500	65	27	28	359	1,551	D2	1,847,672	98	57,088	3%
P40	2	FFPE	2014	500	68	27	28	376	571	D2	2,233,814	98	42,301	2%
P41	1	FFPE	2015	380	nd	27	30	333	nd	D1	1,617,424	97	67,491	4%
P42	2	PE	na	200	53	24	26	401	2,352	D2	2,769,332	98	599,272	22%
Control Samples														
KM12[a]	2	cell line	na	50	96	24	26	487	1,978	D2	2,155,384	96	253,719	12%
KM12[a]	2	cell line	na	10	96	26	28	460	2,625	D2	2,329,356	96	148,920	6%
P34_S1[a]	2	Frozen	na	50	80	23	24	436	3,338	D2	1,925,044	96	482,731	25%
P34_S1[a]	2	Frozen	na	10	81	25	28	426	6,751	D2	1,979,056	96	188,064	10%
P42[a]	2	PE	na	50	51	26	28	404	2,814	D2	1,952,360	96	139,801	7%
P42[a]	2	PE	na	10	45	27	28	462	474	D2	2,052,066	96	49,665	2%
P31[b]	2	FFPE	2014	500	66	26	28	387	1,229	D2	2,120,348	98	66,964	3%
P15[b]	2	FFPE	2014	500	43	25	27	372	1,051	D2	1,731,948	98	73,397	4%
P34_S1[b]	2	Frozen	na	200	82	26	28	407	2,611	D2	1,697,218	98	90,148	5%
P25[C]	1	FFPE	2015	na	na	na	na	na	na	D1	532,946	98	86,792	16%
P03[C]	1	FFPE	2015	na	na	na	na	na	na	D1	332,448	98	22,639	7%
P17[C]	1	FFPE	2014	na	na	na	na	na	na	D1	315,038	98	79,759	25%
P29[C]	1	FFPE	2014	na	na	na	na	na	na	D1	252,738	98	26,561	11%
P15[C]	1	FFPE	2014	na	na	na	na	na	na	D1	250,314	98	29,124	12%
P34_S1[C]	1	Frozen	na	na	na	na	na	na	na	D1	274,416	98	57,478	21%

* samples of which we lost 1/3 to 1/4 of the RNA accidently during library preparation that were excluded from linear regression analysis by SPSS, control samples were not analyzed in linear regression analysis by SPSS; [a] samples analyzed with lower input; [b] samples analyzed both in design one and design two; [c] samples of which 1/8 of the required library input was pooled and sequenced; U%: percentage of unique reads; PE: pleural effusion; FFPE: formalin-fixed paraffin-embedded tissue; na: not applicable; nd: not done.

Table S2. Overview of RNA-based ddPCR results.

Sample ID	Origin	DV 200	MD Variant		NGS Result			ddPCR Validation				
			Gene	Amino Acid Change	Mutant Reads	Total Reads	VAF	Input RNA (ng)	Mutant Copies	Wild Type Copies	Fractional Abundance	Wells
Variants Detected by Our All-In-One Assay												
A549	cell line	na	*KRAS*	p.G12S	512	513	100%	1	380	0	100%	2
H1650	cell line	na	*EGFR*	E19 DEL	72	98	73%	1	2350	1110	68%	2
H1975	cell line	na	*EGFR*	p.L858R	564	684	82%	1	1988	402	83%	2
H1975	cell line	na	*EGFR*	p.T790M	347	425	82%	2	4831	996	83%	1
HCT116	cell line	na	*KRAS*	p.G13D	223	456	49%	1	259	217	54%	2
KOPN-8	cell line	99	*KRAS*	p.G12D	99	177	56%	1	268	283	49%	2
P01	PE	90	*KRAS*	p.G12D	14	111	13%	8	64	364	15%	1
P03	FFPE	na	*KRAS*	p.G12A	8	8	100%	8	10	0	100%	1
P06	FFPE	37	*EGFR*	E19 DEL	20	28	71%	5	527	55	91%	3
P15	FFPE	40	*EGFR*	E19 DEL	119	143	83%	5	629	165	79%	3
P17	FFPE	57	*EGFR*	E19 DEL*	311	373	83%	8	1540	203	88%	2
P17	FFPE	57	*EGFR*	p.T790M	62	127	49%	6	1420	614	70%	1
P22	FFPE	69	*EGFR*	E19 deletion	13	38	34%	8	186	167	53%	2
P39	FFPE	65	*KRAS*	p.G12D	8	12	67%	8	25	23	53%	1
Variants Not Detected by Our All-In-One Assay												
P05	PE	17	*EGFR*	E19 DEL	0	0	na	100	12	9	57%	4
P06	FFPE	37	*EGFR*	p.T790M	0	0	na	324	21	188	10%	3
P23	FFPE	44	*KRAS*	p.G12C	0	0	na	25	19	8	71%	2
P26	FFPE	na	*EGFR*	p.L858R	0	0	na	370	177	422	30%	4
P32	FFPE	32	*KRAS*	p.G12D	0	0	na	497	0	8	0%	3
P40	FFPE	68	*KRAS*	p.G12F	0	0	na	30	95	68	59%	1

* Read counts for EGFR exon 19 are based on IGV; MD: molecular diagnostic; VAF: Variant allele frequency; PE: pleural effusion; FFPE: formalin-fixed paraffin-embedded tissue; na: not analyzed; E19 DEL: exon 19 deletion**.**

Table S3. Overview of NanoString results on samples with expected fusions.

Sample ID	Origin	MD*	Fusion Transcript by RNA-Based Assay	Nanostring Validation
P07	FFPE	*ALK*	not detected	Positive
P08	FFPE	*ALK*	KIF5B_E24-ALK_E20	Positive
P08	FFPE	*RET*	not detected	Negative
P11	FFPE	*RET*	KIF5B_E15-RET_E12	Positive
P18	FFPE	*ALK*	EML4_E6-ALK_E20	Positive
P34_S1	FFPE	*ALK*	EML4_E6-ALK_E20	Positive
P38	FFPE	*ROS1*	EZR_E10-ROS1_E34	Positive

* FISH break and/or IHC positive cases as reported by the molecular diagnostics.

Table S4. Progression free survival of ALK positive patients.

Patient ID	FISH	IHC	Fusion Transcript	TKI	PFS
P07	+	+	EML4_E6-ALK_E20*	crizotinib	14
P08	+	+	KIF5B_E24-ALK_E20	ceritinib	19
P13	+	nd	EML4_E6-ALK_E20	crizotinib	8
P14	+	nd	DCTN1_E26-ALK_E20	crizotinib /alectinib	no response
P18	+	+	EML4_E6-ALK_E20	crizotinib	9
P33	+	+	EML4_E6-ALK_E20	crizotinib	6
P34_S1	+	+	EML4_E6-ALK_E20	crizotinib	15
P34_S2	+	+	EML4_E6-ALK_E20	ceritinib	12
P36	+	+	EML4_E6-ALK_E18	crizotinib	24
P42	+	+	MPRIP_E21-ALK_E20	crizotinib	8

* fusion gene detected by NanoString; PFS: progression free survival; TKI: Tyrosine Kinase Inhibitor; nd: not done

Table S5. Overview of variant calling for samples sequenced with low and normal library input.

Sample ID	Gene	Variant	Standard Library Input			1/8 Library Input		
			Altered Reads/ Splitting Reads	Total Reads/ Spanning Reads	MAF	Altered Reads/ Splitting Reads	Total Reads/ Spanning Reads	MAF
P03	*KRAS*	p.G12A	8	8	100%	4	4	100%
P15	*EGFR*	E19 deletion	81	110	74%	49	58	84%
P15	*EGFR*	p.T790M	22	88	25%	13	50	26%
P17	*EGFR*	E19 deletion	123	143	86%	175	198	88%
P17	*EGFR*	p.T790M	62	127	49%	92	141	65%
P25	*BRAF*	p.V600E	33	46	72%	9	18	50%
P34_S1	*ALK*	fusion	62	41		7	3	

Table S6. Overview of variant calling for samples with normal and low RNA input.

Sample ID	Gene	Input	Fusion (5′ gene-3′ gene)	5' Gene Count-3' Gene Count
KM12	*NTRK1*	200	TPM3_E7-NTRK1_E9	188, 87
KM12	*NTRK1*	50	TPM3_E7-NTRK1_E9	132, 22
KM12	*NTRK1*	10	TPM3_E7-NTRK1_E9	88, 26
P34_S1	*ALK*	200	EML4_E6-ALK_E20	6, 2
P34_S1	*ALK*	50	EML4_E6-ALK_E20	16, 23
P34_S1	*ALK*	10	EML4_E6-ALK_E20	9, 10
P42	*ALK*	200	MRPIP_E21-ALK_E20	8, 20
P42	*ALK*	50	MRPIP_E21-ALK_E20	1, 6
P42	*ALK*	10	MRPIP_E21-ALK_E20	0, 6

Table S7. Overview of variant calling results in relation to QC data.

Sample ID	Variant 1 (*)	Variant 2	Variant 3	Unique reads	RNA input (ng)	Origin	Year of FFPE	DV200	Pass QC **
A549	*KRAS* G12S:+			284,952	200	cell line	na	nd	na
H1299	*NRAS* Q61K:+			321,756	121	cell line	na	nd	na
H1650	*EGFR* E746_A750del:+			97,845	200	cell line	na	nd	na
H1975	*EGFR* L858R :+	*EGFR* T790M :+		235,397	200	cell line	na	nd	na
H820	*EGFR* E746_A750del:+	*EGFR* T790M :+		618,358	200	cell line	na	99	Yes
KOPN-8	*KRAS* G12D:+			201,459	200	cell line	na	99	Yes
HCT116	*KRAS* G13D:+	*PIK3CA* H1047R:+		289,423	188	cell line	na	nd	na
H596	*PIK3CA* E545K:+	*MET* E14 skipping:+		1,262,964	200	cell line	na	98	Yes
Hs746T	*MET* E14 skipping:+			639,210	200	cell line	na	97	Yes
H2228	*ALK* fusion:+			222,701	200	cell line	na	nd	na
KM12	*NTRK1* fusion:+			792,640	200	cell line	na	94	Yes
P18	*ALK* fusion:+			466,598	200	Frozen	na	86	Yes
P34_S1	*ALK* fusion:+			455,287	160	Frozen	na	82	Yes
P35	*AKT1* E17K:+	*BRAF* V600E :+		1,276,594	200	PE	na	81	Yes
P05	*EGFR* E746_A750del:-			167,871	200	PE	na	17	No
P04_S2	*EGFR* L858R:+			1,331,315	200	PE	na	88	Yes
P01	*KRAS* G12D:+			199,647	137	PE	na	90	Yes
P14	*ALK* fusion:+			600,434	200	PE	na	86	Yes
P36	*ALK* fusion:+			1,037,592	200	PE	na	93	Yes
P37	*ROS1* D2033N:+	*ROS1* fusion:+		232,418	200	PE	na	21	No
P42	*ALK* fusion:+			599,272	200	PE	na	53	Yes
P13	*ALK* I1171N:+	*ALK* G1269A :+	*ALK* fusion:+	1,297,907	200	PE	na	89	Yes
P25	*BRAF* V600E:+			139,373	500	FFPE	2015	71	Yes
P15	*EGFR* E746_A750del:+	*EGFR* T790M:+		79,865	304	FFPE	2014	40	Yes
P17	*EGFR* E746_A750del:+	*EGFR* T790M:+		79,759	195	FFPE	2014	57	Yes
P22	*EGFR* E746_A750del:+			86,309	500	FFPE	2014	69	Yes
P06	*EGFR* L747_P753delinsS:+	*EGFR* T790M:-		23,351	500	FFPE	2016	37	No
P26	*EGFR* L858R:-	*PIK3CA* E542K:-		59,613	500	FFPE	2014	nd	na
P04_S1	*EGFR* L858R:+			221,944	500	FFPE	2017	26	No
P03	*KRAS* G12A:+			67,381	500	FFPE	2015	nd	na
P28	*KRAS* G12A:+			88,560	380	FFPE	2017	38	Yes
P23	*KRAS* G12C:-			19,638	500	FFPE	2015	44	No
P32	*KRAS* G12D:-			4,915	500	FFPE	2011	32	No
P39	*KRAS* G12D:+			57,088	500	FFPE	2014	65	Yes

P40	*KRAS* G12F:-		42,301	500	FFPE	2014	68	No
P31	*KRAS* Q61H:+		211,390	500	FFPE	2014	66	Yes
P02	*PIK3CA* H1047L:+		36,593	500	FFPE	2015	51	No
P21	*MET* E14 skipping:+		80,234	432	FFPE	2017	34	Yes
P07	*ALK* L1196M:+	*ALK* Fusion: -	41,696	500	FFPE	2016	65	No
P08	*ALK* fusion:+	(*RET* fusion)	178,860	500	FFPE	2016	67	Yes
P33	*ALK* fusion:+		21,902	500	FFPE	2012	58	No
P34_S1	*ALK* fusion:+		78,515	500	FFPE	2013	76	Yes
P34_S2	*ALK* fusion:+		145,018	500	FFPE	2013	70	Yes
P11	*RET* fusion:+		79,747	500	FFPE	2017	81	Yes
P38	*ROS1* fusion:+		107,167	500	FFPE	2014	55	yes
P41	*ROS1* fusion:+		67,491	380	FFPE	2015	nd	na

* variants observed are indicated in black and marked with +; variants not observed are indicated in red and marked with - ; **Pass QC: passed assay quality control; Variants between brackets are true negatives. PE: pleural effusion; FFPE: formalin-fixed paraffin-embedded; na: not applicable; nd: not determined**.**

Table S8. Linear Regression analysis to identify variables associated with percentage of unique reads.

Variable	Univariable Analysis		Multivariable Analysis	
	**ß (95% Confidence)**	***p* Value**	**ß (95% Confidence)**	***p* Value**
Design Version	0.063 (0.003, 0.123)	0.039	0.037 (−0.022, 0.096)	0.215
Material Type	0.213 (0.15, 0.275)	0.000	0.133 (0.016, 0.249)	0.027
RNA Input	−0.001 (−0.001, 0)	0.000	−0.0001 (−0.0004, 0.0003)	0.604
DV200	0.004 (0.003, 0.006)	0.000	0.002 (0, 0.004)	0.019
PCR Cycles	−0.03 (−0.041, −0.019)	0.000	−0.006 (−0.023, 0.011)	0.488

Table S9. Number of landing probes per gene in the three designs.

Category	Gene	Landing Probe Count		
		Design 1	Design 2	Design 3
mutation	*AKT1*		2	1
mutation	*BRAF*	7	4	7
mutation	*DDR2*		5	2
mutation	*EGFR*	10	11	10
mutation	*ERBB2*		5	5
mutation	*HRAS*		6	7
mutation	*JAK2*		2	17
mutation	*KIT*		15	14
mutation	*KRAS*	4	6	7
mutation	*MAP2K1*		13	13
mutation	*NRAS*	4	11	8
mutation	*PDGFRA*		8	8
mutation	*PIK3CA*	6	8	9
mutation	*POLE*		5	12
mutation	*STK11*	5		
mutation	*STAT3*			12
mutation	*TP53*			27
exon skipping	*MET*	111	14	11
fusion	*ALK*	70	105	70
fusion	*RET*	60	98	58
fusion	*ROS1*	92	140	140
fusion	*KMT2A*		14	
fusion	*MLLT1*		14	
fusion	*NRG1*			34
fusion	*NTRK1*		20	46
fusion partner	*ATP1B1*			34
fusion partner	*CCDC6*	48	86	24
fusion partner	*CD74*	24	34	26
fusion partner	*EML4*	62	86	50
fusion partner	*EZR*	36	54	38
fusion partner	*HIP1*	87	116	66
fusion partner	*KIF5B*	70	100	62
fusion partner	*SDC4*	22	38	12
fusion partner	*SLC34A2*	44	65	32
expression	*BRCA1*			8
expression	*BRCA2*			8
expression	*HPRT1*		6	

Category	Gene	Landing Probe Count		
		Design 1	Design 2	Design 3
expression	*MPRIP*		10	10
expression	*MYC*	18	4	4
expression	*MYCN*	21	4	
expression	*POLR2A*		4	12
expression	*PXDNL*			8
expression	*RYR1*			8
expression	*SCN8A*			6
expression	*SLIT3*			12
expression	*FGFR1*	60	6	4
expression	*FGFR3*	47	6	6
expression	*RICTOR*			12
gender control	*AMELY*		2	2
housekeeping	*B2M*			14
housekeeping	*CD274*			8
housekeeping	*GAPDH*	8		
housekeeping	*HRPT1*	59		
housekeeping	*GP9*		4	4
housekeeping	*GUSB*			9
housekeeping	*HAVCR2*			6
housekeeping	*HMBS*			4
housekeeping	*IPO8*		4	6
housekeeping	*PAPPA2*			22
housekeeping	*PPIA*			4
housekeeping	*PTPRC*		8	6
housekeeping	*RP2*	42	6	6
housekeeping	*RPS9*			8
housekeeping	*TBP*			11
housekeeping	*UBC*			2
housekeeping	*YWHAZ*			10

© 2020 by the authors. Licensee MDPI, Basel, Switzerland. This article is an open access article distributed under the terms and conditions of the Creative Commons Attribution (CC BY) license (http://creativecommons.org/licenses/by/4.0/).
